# Coverage and Timing of Children's Vaccination: An Evaluation of the Expanded Programme on Immunisation in The Gambia

**DOI:** 10.1371/journal.pone.0107280

**Published:** 2014-09-18

**Authors:** Susana Scott, Aderonke Odutola, Grant Mackenzie, Tony Fulford, Muhammed O. Afolabi, Yamundow Lowe Jallow, Momodou Jasseh, David Jeffries, Bai Lamin Dondeh, Stephen R. C. Howie, Umberto D'Alessandro

**Affiliations:** 1 Medical Research Council Unit, Fajara, The Gambia; 2 London School of Hygiene and Tropical Medicine, London, United Kingdom; 3 Ministry of Health and Social Welfare Government of The Gambia, The Quadrangle, Banjul, The Gambia; University of Cambridge, United Kingdom

## Abstract

**Objective:**

To evaluate the coverage and timeliness of the Expanded Programme on Immunisation (EPI) in The Gambia.

**Methods:**

Vaccination data were obtained between January 2005 and December 2012 from the Farafenni Health and Demographic Surveillance System (FHDSS), the Basse Health and Demographic Surveillance System (BHDSS), the Kiang West Demographic surveillance system (KWDSS), a cluster survey in the more urban Western Health Region (WR) and a cross sectional study in four clinics in the semi-urban Greater Banjul area of WR. Kaplan-Meier survival function was used to estimate the proportion vaccinated by age and to assess timeliness to vaccination.

**Findings:**

BCG vaccine uptake was over 95% in all regions. Coverage of DPT1 ranged from 93.2% in BHDSS to 99.8% in the WR. Coverage decreased with increasing number of DPT doses; DPT3 coverage ranged from 81.7% in BHDSS to 99.0% in WR. Measles vaccination coverage ranged from 83.3% in BHDSS to 97.0% in WR. DPT4 booster coverage was low and ranged from 43.9% in the WR to 82.8% in KWDSS. Across all regions, delaying on previous vaccinations increased the likelihood of being delayed for the subsequent vaccination.

**Conclusions:**

The Gambia health system achieves high vaccine coverage in the first year of life. However, there continues to be a delay to vaccination which may impact on the introduction of new vaccines. Examples of effectively functioning EPI programmes such as The Gambia one may well be important models for other low income countries struggling to achieve high routine vaccination coverage.

## Introduction

As the 2015 target date for the Millennium Development Goals (MDGs) approaches, there is the need to evaluate health programmes and assess how effective they are. The Expanded Programme on Immunisation (EPI) has been one of the most successful global childhood programmes, reducing mortality and morbidity from vaccine-preventable diseases and providing opportunities for other interventions. EPI vaccination visits (usually at birth, 6, 10 and 14 weeks and 9 months of age) are crucial contact points between a child and the health system, where vaccines and other essential health care interventions like Long-Lasting Insecticidal Nets (LLINs), de-worming, vitamin A, and growth assessments, are given [1].

Despite the EPI's impact on child health the burden of vaccine-preventable diseases, particularly in the most vulnerable infants, remains high [2]. Access to health care, including vaccination, remains low in many parts of world [3]. The evaluation and optimisation of vaccination programmes were identified as priorities at the Strategic Advisory Group of Experts (SAGE) meeting in 2005, which noted that one size does not fit all. Immunisation uptake varies across the world and its impact varies according to differences in epidemiology, health infrastructure and resources [4]. Though vaccination coverage is an important measure of access, timing of vaccination is equally important as immunisation programmes can be successful only if children are protected prior to exposure. Timing also has implications for the introduction of other vaccines; for example, until recently there was an age restriction on the administration of rotavirus vaccine, thus limiting its use in many countries [5], [6]. Delayed administration of vaccines is common and if the rotavirus vaccine schedule was to adhere to the Diphtheria-Pertussis-Tetanus (DPT) vaccine schedule, more than 30% of children would be past the recommended age at their first DPT dose [7]. Vaccination timeliness varies widely between and within countries, and this is masked by coverage estimates [8]. There have been few studies on vaccine uptake and predictors for delayed administration in sub-Saharan Africa [9]–[11]. However, each country needs to evaluate its own vaccine programmes for making evidence-based decisions on vaccine schedules, assessing the suitability of new vaccines, and monitoring both the local epidemiology of infection/disease and the available financial resources. Following a yellow fever epidemic in 1978 in the Upper River Divisions, the EPI was initiated in The Gambia in May 1979 [12]. The initial vaccines were BCG, Diphtheria-Tetanus- whole cell Pertussis (DTwP), measles, oral polio (OPV) and yellow fever. Hepatitis B (HepB) vaccine was phased into the EPI between 1986 and 1990. Haemophilus influenzae type b (Hib) vaccine was introduced in 1997 and in 2009 the pentavalent vaccine (DwPT-HepB-Hib) replaced the DTwP-HepB quadrivalent and monovalent HepB and Hib vaccines. Pneumococcal conjugate vaccine (PCV) was also introduced in 2009. The Gambia, unlike most other sub-Saharan African countries, also provides a booster dose of DPT at 18 months of age. Here we use data sets across The Gambia to evaluate the coverage and timeliness of the national EPI programme.

## Methods

### Ethics statement

Approval was obtained from Gambia Government/Medical Research Council Joint Ethics Committee.

### Study populations and data collection

**Figure 1:**
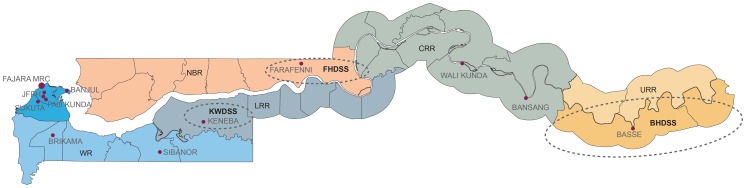
Map of The Gambia. WCR: Western Coastal Region; LRR: Lower River Region; CRR: Central River Region; URR: Upper River Region. Dashed circles represent the demographic surveillance sites: Farafenni Health and Demographic Surveillance System (FHDSS), Basse Health and Demographic Surveillance System (BHDSS), and Kiang West Demographic surveillance system (KWDSS).

The Gambia is a small West African country with a population of under 2 million, an annual gross national income per capita of $1,750 and an under five mortality rate of 101 per 1000 live births (http://www.who.int/countries/gmb/en/). Data sources were obtained from four different geographical regions; Western health Region (WR), North Bank Region (NBR), Lower River Region (LRR) and Upper River Region (URR) (Figure 1). The study design and definition of variables for each of the study sites are summarised in Table 1.

**Table 1:** General characteristics of the study population and study sites to evaluate the Expanded Programme on Immunisation in the Gambia between January 2005 and December 2012.

Study site	Population	Study design	Total population surveyed	Date of data collection period	Age range for data collection
North bank region	42 rural villages and Farafenni town + satellite villages within 5 km radius, North Bank Region	HDSS	7,182	Jan 2005–Aug 2012	<5 years
Upper River Region	Southern Bank of the Upper River Region	HDSS	34,524	Jan 2005–Dec 2012	<5 years
Lower river region	West Kiang regions	DSS and KeMRES	512	Jan 2005– Feb 2012	<5 years
Western health region	Capital of Banjul, the Greater Banjul area and the Western Coastal Region	EPI cluster survey	923	June 2010	12–23 months
Greater Banjul health facilities	Fajikunda, Serrekunda, JFPH and Sukuta HealthClinics	Cross-sectional	1,403	Jan 2010–Dec 2011	>9months -5years

HDSS: Health and Demographic surveillance system; DSS: demographic surveillance system; KeMRES: Keneba Electronic Medical Records System; EPI: Expanded Programme of Immunisation.

Vaccination data were obtained from three rural demographic surveillance sites in The Gambia; the Farafenni Health and Demographic Surveillance System (FHDSS) [13], [14], the Basse Health and Demographic Surveillance System (BHDSS) [15]–[17], and the Kiang West Demographic surveillance system (KWDSS) [18] (Figure 1). The methods have been already published elsewhere [13]–[18]. Briefly, each household within the surveillance area is visited every 3–4 months to collect information on births, deaths, in and out migrations, pregnancies, marriages and the vaccination status of all under-five children.

In June 2010, vaccine coverage in the Western Health Region (WR) was assessed in 12–23 month olds using the cluster survey technique recommended by the World Health Organization [19], [20].

Between January 2010 and December 2011, a cross sectional study was carried out in four clinics in the semi-urban Greater Banjul area of WR. All children aged 9 to 60 months attending the well-baby clinics at the Fajikunda, Sukuta, and Serrekunda health centres and Jammeh Foundation for Peace Hospital (JFPH), and who had not been involved in previous vaccine trials were invited to participate. A questionnaire on vaccination status and socio-demographic characteristics was administered to the caregivers.

### Statistical analysis

Vaccine coverage for the primary series (BCG, three doses of DPT (DPT1, DPT2 and DPT3) and measles vaccines) and the booster dose of DPT administered at 18 months were calculated. A child was considered to have been vaccinated if a vaccination date was recorded and not vaccinated if no vaccination date was recorded. The proportion vaccinated was calculated for BCG and measles vaccines and by the number of DPT vaccine doses as the number of vaccinated children divided by the total number of surveyed children eligible for vaccination at time of interview according to the vaccination schedule (Table 2). A DPT booster dose is defined as one dose of DPT given at least one year after the last DPT dose in the primary series. Median age and interquartile ranges (IQR) for each dose of BCG, measles and DPT were calculated.

**Table 2:** Age for WHO recommended routine vaccinations in the Gambia.

	Schedule 1	Schedule 2
Vaccinations	Current EPI routine Gambian Schedule	Adapted WHO recommended age range for vaccination [7]
BCG/OPV/HebB	Birth	Birth-8 weeks
DTwP-Hib-HepB/PCV/OPV	2 months	6 weeks–3 months
DTwP-Hib-HepB/PCV/OPV	3 months	10 weeks–5 months
DTwP-Hib-HepB/PCV/OPV	4 months	14 weeks–7 months
Measles/Yellow Fever/OPV	9 months	38 weeks–12 months
DPT/OPV	18 months	15 months–24 months

Yearly birth cohorts were used (instead of calendar year) so that denominators and numerators were of the same group and to take account of delayed vaccinations that went across calendar years. For each vaccine, the proportion vaccinated by age was estimated using 1 minus the Kaplan-Meier survival function [21], [22]. Censoring for each child occurred at date of vaccination, or if not vaccinated, at the date of interview.

To assess timeliness of vaccination we used the current Gambia EPI schedule (schedule 1) and an adapted WHO recommended age range (schedule 2) (Table 2) [7]. The Kaplan-Meier method was used to assess timeliness of each vaccination schedule. An event was defined as having been vaccinated before the upper limit of age range for each specific vaccine schedule. Those who were vaccinated after these limits were considered failures. Cox regression analysis was used to examine factors associated with delayed vaccination for each vaccine. These factors include year of birth, gender, ethnicity and number of previous delayed vaccinations. Covariates were retained in the model if associations were observed at the p<0.05 level and/or if they altered substantially the associations of other effect variables in multivariable analysis. Statistical analyses were performed using STATA 12.0 statistical software (StataCorp LP, USA, http://www.stata.com).

## Results

A total of 44,544 children were included in this analysis; 77.5% from URR, 16.1% from NBR, 1.2% from LRR, 2.1% from WR and 3.2% from the Greater Banjul health facilities. As the sample sizes from each geographical region varied substantially results are presented by study site.

### Vaccination coverage

**Figure 2:**
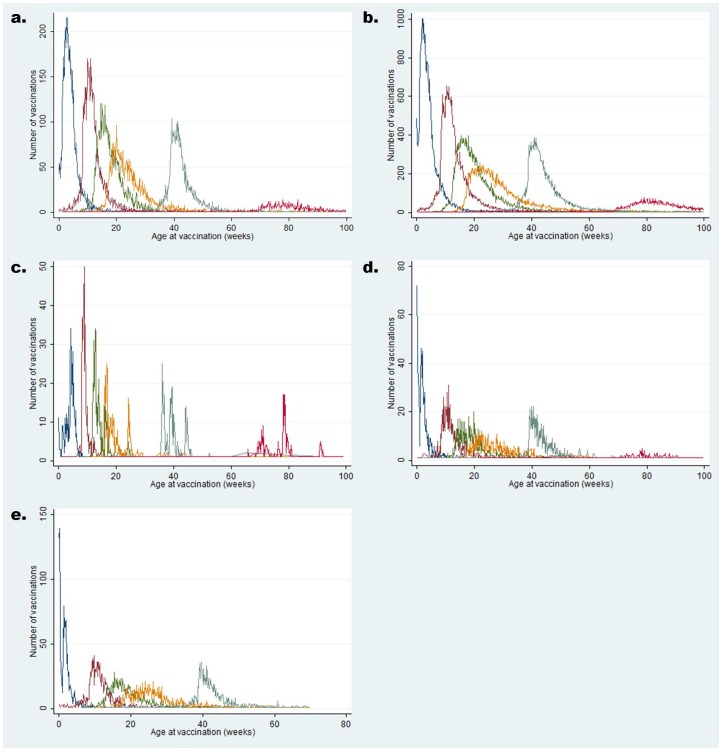
Frequency distribution of number of vaccinations received by age at vaccination (in weeks) for each of the study sites: a) North Bank region (2005–2012), b) South bank of the Upper River Region (2005–2012), c) West Kiang, Lower River Region (2005–2012), d) Western Health Region (June 2010), e) Greater Banjul Health facilities (2010–2011). Blue line: Proportion vaccinated with BCG vaccine. Brown line: Proportion vaccinated with 1 dose of DPT vaccine. Green line: Proportion vaccinated with 2 doses of DPT vaccine. Orange line: Proportion vaccinated with 3 doses of DPT vaccine. Blue/Green line: Proportion vaccinated with measles vaccine. Red line: Proportion vaccinated with DPT booster.

**Figure 3:**
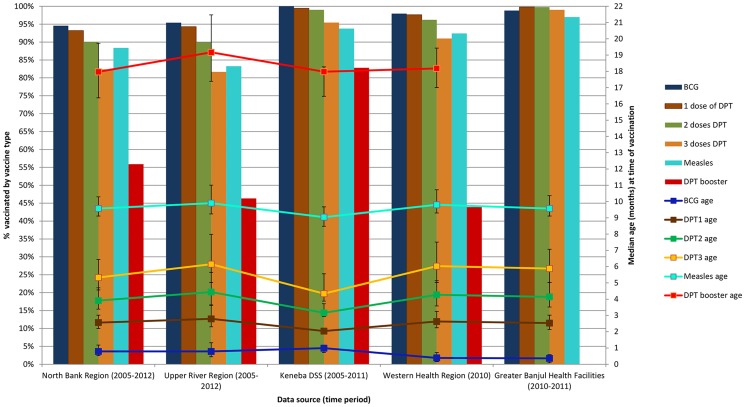
Proportion of eligible children vaccinated and median age (in months) by vaccine type and by region in The Gambia. Blue bars: proportion who received BCG on time. Brown bars: proportion who received DPT1 on time. Green bars: proportion who received DPT2 on time. Orange bars: proportion who received DPT2 on time. Turquoise bars: proportion who received measles vaccine on time. Red bars: proportion who received DPT booster on time. Blue line: Median age (in months) for BCG. Brown line: Median age (in months) for DPT1. Green line: Median age (in months) for DPT2. Orange line: Median age (in months) for DPT3. Turquoise line: Median age (in months) for measles vaccine. Red line: Median age (in months) for DPT booster. Vertical bars: Interquartilte range in months of age at vaccination.

Vaccination coverage and age distribution at time of vaccination were similar across all geographical regions (Figures 2 and 3).

BCG uptake was high with over 95% coverage in all areas. The median age of BCG vaccination was just under 2 weeks in the urban WR, and around 3–4 weeks in the rural areas of NBR, URR and LRR.

A high coverage of the primary series was achieved in all geographical regions. Coverage of the first dose of DPT ranged from 93.2% in NBR to 99.8% in the Greater Banjul health facilities. Coverage for two doses ranged from 90.1% in URR to 99.8% in the Greater Banjul health facilities. A slightly lower coverage of three doses of DPT was observed across all regions; ranging from 81.7% in URR to 99.0% in Greater Banjul clinics. The median age that DPT doses were received ranged between 2.5 (IQR 2.1–3.0) and 2.8 (IQR 2.3–3.6) months for DPT1, between 3.9 (IQR3.4–4.7) and 4.4 (IQR3.7–5.7) months for DPT2 and between 5.3 (IQR 4.6–6.4) and 6.2 (IQR 5.0–8.0) months of age for DPT3, with West Kiang being closest to the prescribed schedule. Measles vaccination coverage ranged from 83.3% in URR to 97.0% in Greater Banjul clinics. The median age for measles vaccination was similar across all regions, ranging from 9.0 (IQR 8.5–9.6) to 9.9 (IQR 9.2–11.0) months. The coverage for the DPT booster dose was much lower compared to those of the primary series; ranging from 43.9% in the WR to 82.8% in West Kiang. The median age ranged between 18.0 (IQR 16.4–19.7) and 19.2 (IQR 17.4–21.5) months.

### Proportion vaccinated by age

**Figure 4:**
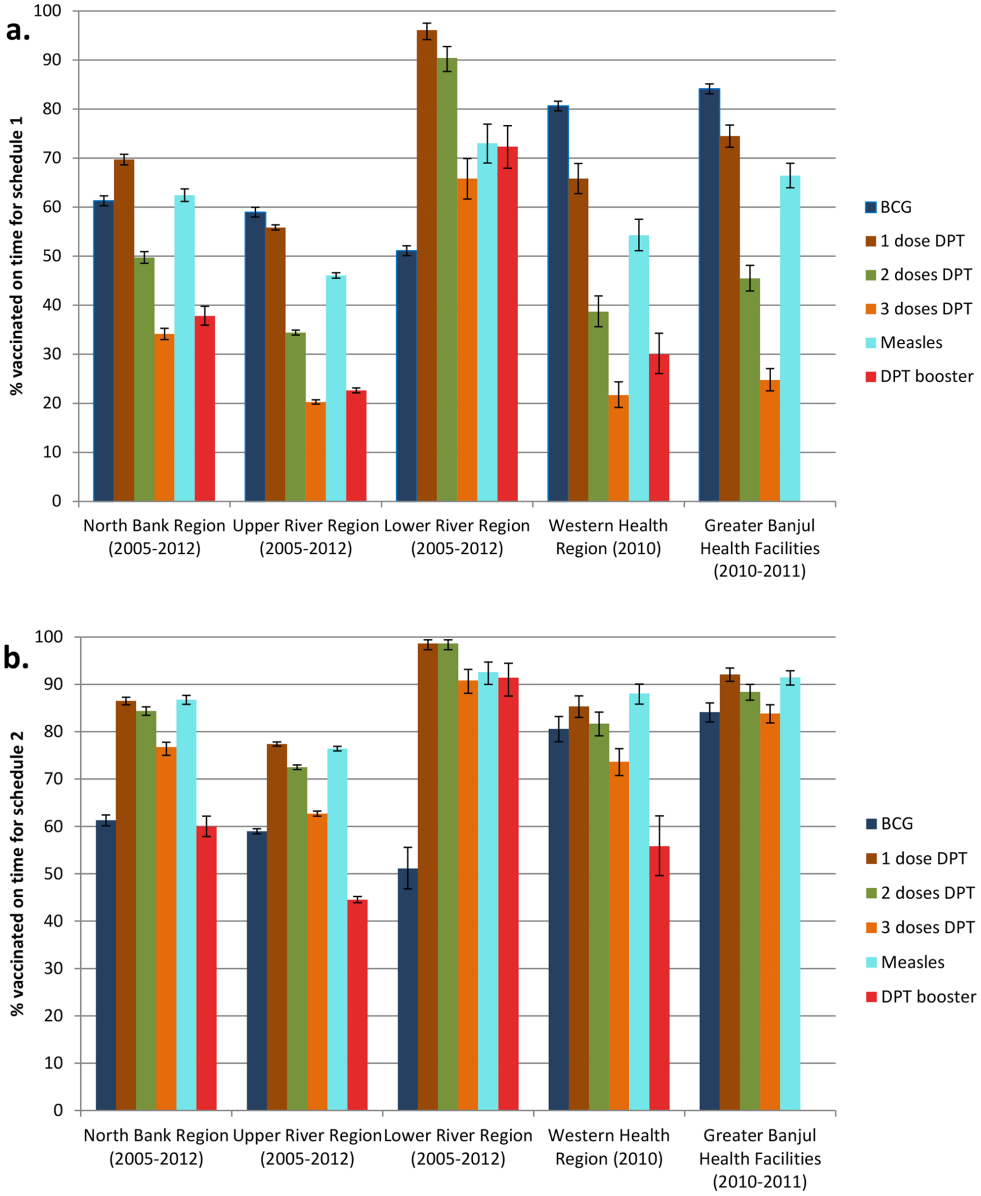
Estimated proportion vaccinated on time by age (months) by vaccine for each geographical region in the Gambia for a) schedule 1 and b) schedule 2. Blue bars: proportion who received BCG on time. Brown bars: proportion who received DPT1 on time. Green bars: proportion who received DPT2 on time. Orange bars: proportion who received DPT3 on time. Turquoise bars: proportion who received measles vaccine on time. Red bars: proportion who received DPT booster on time. Vertical bars: 95% confidence intervals.

The proportion vaccinated by age for each vaccine was estimated using 1 minus the Kaplan-Meier survival function (Figure 4). Further details for each specific geographical region are shown in Tables S1a-e in File S1. By the end of one month of life, approximately 50–60% of children were estimated to have been vaccinated with BCG in the rural areas of NBR, URR and LRR. Over 80% of children in the WR had received BCG vaccination by the end of their first month.

Using schedule 1, the proportion vaccinated on time with DPT1 ranged from 55.8% (95% CI: 55.31–56.36) in URR to 96.1% (95% CI: 94.1–97.5) in West Kiang, LRR. The proportion vaccinated on time with DPT2 ranged from 34.4% (95% CI: 33.90–34.92) in URR to 90.1% (95% CI: 87.64–92.75) in West Kiang, LRR. For DPT3, only 20.3% (95% CI: 19.84–20.70) of URR children were vaccinated on time compared to 65.8% (95 CI: 61.67–69.92) in West Kiang. For measles vaccine, coverage by 10 months of age ranged from 46.1% (95% CI: 45.51–46.61) in URR to 73.0% (95% CI: 69.01–76.91) in West Kiang. The coverage of the DPT booster dose by 19 months of age was less than 40% in children from URR, NBR and the Western regions. 72.3% (95% CI: 67.94–76.59) of children vaccinated with the DPT booster dose in West Kiang, did so on time (Figure 4a).

Using schedule 2, the patterns on time vaccination coverage was similar to schedule 1 but at higher coverage (Figure 4b).

### Predictors for delayed vaccinations

Overall, compared to other study sites, a higher proportion of children in the URR region were delayed for all vaccinations (Table 3). Children from West Kiang were more likely to return on time for vaccination. For BCG vaccine, the proportion experiencing delays ranged from 2.0% in West Kiang to 14.6% in URR. An increasing proportion of children were delayed with increasing number of DPT doses for all study sites; ranging from 1.0%–18.8% for DPT1 and increasing to 5.2%–24.8% for DPT3. For those vaccinated with measles vaccine, the proportion who delayed ranged from 1.3% in West Kiang to 19.3% in the Western health region. Very few children in West Kiang were delayed for DPT4 (0.55%) compared with 11% of children in URR.

**Table:** **Table 3.** Proportion delayed to vaccination and median delay by vaccine type and by geographical region in The Gambia.

Geographical region	Vaccine	Total vaccinated (n)	% vaccinated who delay	Median delay^1^ (weeks)	IQR(weeks)
	BCG	6,738	9.26	3.71	(1.14, 12.93)
	DPT1	6,538	8.61	4.00	(1.57, 10.71)
North bank	DPT2	6,163	8.71	4.86	(1.71, 13.86)
Region	DPT3	5,418	10.5	6.71	(2.57, 16.86)
	Measles	4,791	4.97	18.43	(4.93, 35.93)
	DPT-Booster	1,238	3.63	11.79	(4.93, 23.50)
	BCG	32,490	14.6	4.14	(1.43, 14.86)
South bank,	DPT1	32,311	18.83	5.00	(1.86, 13.00)
Upper river	DPT2	30,614	20.56	6.71	(2.57, 16.71)
Region	DPT3	27,554	24.78	10.42	(4.00, 26.71)
	Measles	26,387	10.5	12.50	(4.64, 33.93)
	DPT-Booster	9,737	11.1	16.79	(6.21, 33.79)
	BCG	501	2	0.78	(0.29, 2.71)
	DPT1	509	0.98	1.71	(1.14, 2.00)
West Kiang,	DPT2	505	0.4	14.86	(0.29, 29.43)
Lower river	DPT3	483	5.18	2.57	(1.86, 17.57)
Region	Measles	452	1.33	21.43	(8.36, 46.79)
	DPT-Booster	361	0.55	48.29	(3.64, 92.93)
Western	BCG	832	6.61	4.00	(1.29, 10.29)
Health	DPT1	901	13.1	3.57	(1.29, 8.71)
Region, EPI	DPT2	888	15.32	6.07	(2.71, 11.93)
Cluster	DPT3	840	19.29	5.93	(3.14, 15.14)
Survey	Measles	852	5.75	4.64	(2.64, 13.50)
Greater	BCG	1,254	7.89	7.71	(3.00, 16.00)
Banjul	DPT1	1,400	7.93	4.14	(1.71, 10.00)
Health	DPT2	1,400	11.86	4.86	(1.43, 9.00)
Clinics	DPT3	1,389	15.55	6.29	(2.86, 12.26)
	Measles	1,345	7.06	8.79	(4.21, 25.79)

IQR: Interquartile range in weeks; ^1^Median delay estimated from the upper limit of schedule 2 for each vaccine.

Details of predictors for delay to vaccination for each study site are shown in Tables S2–S6 in File S1. Across all regions, delaying on previous vaccinations increased the likelihood of being delayed for the subsequent vaccination. This association was more strongly observed for the third dose of DPT. Delay to vaccination for all vaccines decreased over time between 2005 and 2010 for the NBR and URR demographic surveillance sites. In NBR and URR regions, Mandinkas were more likely to be vaccinated on time compared to Fulas for BCG and DPT1-3 but not for measles or DPT booster vaccines once adjusted for year of birth and number of previous delayed vaccinations. In the Western Region clinics and West Kiang there was no evidence for an association between ethnic group and being vaccinated on time.

## Discussion

The Gambia health system achieves high vaccine coverage in the first year of life. Vaccination is offered for free and there are mobile services for remote populations. As a result, vaccination coverage is over 95% for BCG, >93% for one dose of DPT, and approximately 90% for 2 doses of DPT. Coverage of the third dose of DPT and measles are lower, particularly in the URR and NBR rural areas [13], as is coverage of the fourth dose of DPT at 18 months of age. Coverage of later doses is often lower as parents are less likely to return to clinics as a child grows, possibly through emphasis on younger siblings [9], and because of a similar health system emphasis on infants.

Children receive DPT1 usually between 2–3 months, DPT2 between 3 and 4.5 months and DPT3 between 4–6 months of age. This observation has important implications, not only for the success of the DPT vaccines but also for the other vaccines delivered at these times points, i.e. PCV, HepB, OPV and Hib *vaccines*. Timeliness also has implications for the introduction of other vaccines. Until recently, due to safety concerns, there was an age restriction on the rotavirus vaccine schedule [5], [6]. Clark and Sanderson examined demographic and health surveys (DHS) in 45 countries (Gambia not included) to estimate vaccination coverage and delays in administration at national level [7]. They observed that if the rotavirus vaccine schedule was to adhere to the DPT schedule then more than 30% of children would be past the recommended age when they were given the first dose of DPT. In this study, we observe that 13.51–22.6% in rural areas and 7.91–14.64% in urban areas would be past the recommended age. Although, this age restriction has now been lifted, reduced delay will further improve safety as well as more infants being protected at an earlier age

We explored the estimated proportion vaccinated by age and predictors for delayed vaccination. These are important indicators for ensuring that vaccines are being delivered before a child is at risk of acquiring infection and also to develop strategies to improve age-appropriate vaccination coverage [22]. The proportion vaccinated on time decreased with increasing return visits for vaccination but increased over time, indicating that programmes are continuing their efforts to reach all children on time. The combined pentavalent and PCV vaccines were introduced in 2009. We observed no change in uptake or increased delays as the health systems adjusted, which is reassuring and consistent with observations seen in Kenya [10].

Previous delay in vaccinations was associated with further delay in later vaccination rather than a reversion to the original schedule. This applied not only to the primary series but also to measles and DPT booster doses, suggesting that this may reflect not only a scheduling knock-on effect but also a tendency for families who delay initially to continue doing so. Early delayers may be a group needing the specific attention of the health staff.

This study had potential weaknesses: The DSS data sets depend on interviewers visiting each home and it is possible that not all households were visited or all records were adequately collected. For NBR, this data set represented 59.8% all births in this region between 2005 and 2012. As noted by Payne and colleagues, those not surveyed were more likely to come from urban areas and have higher educational and wealth levels [13]. In URR and West Kiang, a great effort is placed on visiting all households, and these datasets include all children [15]. Careful consideration was made when deciding what years of data to include for each data set. Although the FDHSS was set up in 1981, a customized enhanced system was designed in 2005 using the MSDE database engine, which is part of MS Access. This meant that recording of events were better monitored. Data previous to 2005 were entered retrospectively and a decision was thus made to only include the prospectively collected data from 2005 onwards. This prospective data collection system was also used for the BDHSS since its inception and from 2005 onwards for the KWDSS. Thus, the data collection methods are similar across the years and we are confident that they are of similar quality. The major strength of the DSS data sets is that they include a prospective, rigorous and frequent data collection process. The fact that timelines and uptake patterns are remarkably similar across all the varying data sets and geographical regions further strengthens the quality of data.

We did not take into account mortality and some children may have died before being eligible for vaccination. To include these children may have underestimated vaccine coverage and increased the estimate of the magnitude of delay. The design of this work also assumes that those with a vaccine date had been vaccinated and those with missing vaccination dates were not vaccinated. The presence of a vaccination card may be associated with a greater probability of being vaccinated [22] and to exclude those with no card may have resulted in underestimating vaccine delay. In all data sets over 97% of children had a health card. We used 1 minus the Kaplan-Meier function to estimate the proportion vaccinated by age consistent with previous studies [7], [9]–[11]. This method is an easy and useful way of visualizing the vaccination uptake over time (or age) and provides estimates of the proportion vaccinated at a given age, which may be useful in assessing the performance of vaccination programmes in reaching their targets [22]. However, this method will consistently give higher results than conventional methods due to censoring, which reduces the population at risk at the time point when censoring occurs. Also, as the number of persons under observation decreases with time, the rightward part of the curve becomes unstable [21]. This would not have significantly affected the primary series but caution should be taken when interpreting the delay to DPT4 if the time of interview was close to the vaccination date.

**Figure 5:**
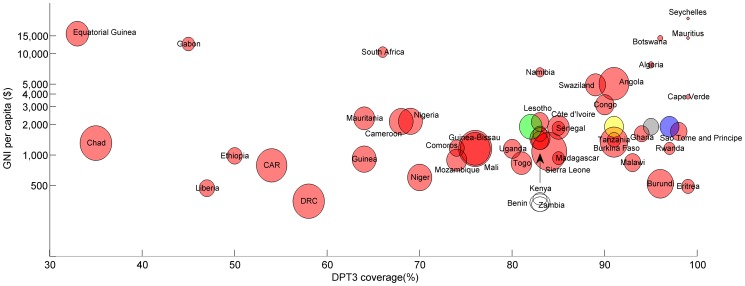
DPT3 coverage, GNI and under-5 mortality rate by countries in the AFRO region. Circle size represents size of under-5 mortality rate. (http://data.worldbank.org/indicator/SH.DYN.MORT). Blue circle: WHO estimates for The Gambia. (http://apps.who.int/immunization_monitoring/globalsummary). Green circle: URR and NBR combined. Grey circle: Western health region. Yellow circle: West Kiang, LRR. GNI: Gross National Income per capita.

EPI was initiated in the Gambia in the same year as the Primary Health Care (PHC) strategy. The primary aim of PHC was “making health care more accessible and affordable to the majority of Gambians” [23]. Maternal and Child health (MCH) services form part of the PHC strategy and include delivering vaccines via static (e.g. health centres and clinics), outreach (regular services in communities more than 8 kms from health facilities) and mobile (vaccination teams in defined target populations) health services throughout the country [12]. These teams also provide a reliable transport system and ensure the cold chain is maintained. Before 2009, 5 Dalasi (approx US$0.17) was charged for a child health card with all subsequent care freely provided [13]. Thereafter, vaccinations and health care have been free for all under-5 year olds. The Gambia, like many other sub-Saharan countries, is a resource -poor country with high under 5 mortality rate and a low GNI (Figure 5). However, these efforts and a continued strong commitment to reach all children by providing free vaccines and increasing access with mobile units have resulted in an EPI programme in The Gambia working well in terms of coverage and has led to near elimination of Hib disease in The Gambia [24], a dramatic decline in vaccine-type pneumococcal incidence (G Mackenzie, unpublished data), a 95% vaccine efficacy against chronic hepatitis B infection after 24 years of hepatitis B vaccination [25], and Gambia being declared polio free in 2004 [12]. The Decade of Vaccines/Global Vaccine Action Plan have set the target of achieving 90% coverage for the first three doses of DPT by 2015 [26]. The results here are promising but rural areas are still just missing this target. Timeliness could also be improved, particularly for later doses, and interventions such early counselling for delayers may assist this. As we look to and beyond the 2015 Millennium Development Goal deadline, examples of effectively functioning EPI programmes such as The Gambia one may well be important models for other low and middle income countries struggling to achieve high routine vaccination coverage.

## Supporting Information

File S1 **This file contains Table S1–Table S6.** Table S1. Estimated proportion vaccinated by age, vaccine type and geographical region in the Gambia. Table S1a. North bank region. Table S1b. South bank, Upper River Region. Table S1c. West Kiang, Lower River region. Table S1d. Western health region. Table S1e. Greater Banjul Health facilities, DPT booster dose not recorded. Table S2. North bank region: Proportion delayed to vaccination and predictors for delay by vaccine type using the adapted WHO recommended vaccine schedule. Table S2a. BCG, delay if vaccinated after 8 weeks of age. Table S2b. DPT1, delay if vaccinated after 3 months of age. Table S2c. DPT2, delay if vaccinated after 5 months of age. Table S2d. DPT3, delay if vaccinated after 7 months of age. Table S2e. Measles vaccine, delay if vaccinated after 12 months of age. Table S2f. DPT-booster dose, delay if vaccinated after 24 months of age. Table S3. South bank, upper river region. Proportion delayed to vaccination and predictors for delay by vaccine type using the adapted WHO recommended vaccine schedule. Table S3a. BCG, delay if vaccinated after 8 weeks of age. Table S3b. DPT1, delay if vaccinated after 3 months of age. Table S3c. DPT2, delay if vaccinated after 5 months of age. Table S3d. DPT3, delay if vaccinated after 7 months of age. Table S3e. Measles vaccine, delay if vaccinated after 12 months of age. Table S3f. DPT-booster dose, delay if vaccinated after 24 months of age. Table S4. West Kiang, Lower river region. Proportion delayed to vaccination and predictors for delay by vaccine type using the adapted WHO recommended vaccine schedule. Table S4a. BCG, delay if vaccinated after 8 weeks of age. Table S4b. DPT1, delay if vaccinated after 3 months of age. Table S4c. DPT2, delay if vaccinated after 5 months of age. Table S4d. DPT3, delay if vaccinated after 7 months of age. Table S4e. Measles vaccine, delay if vaccinated after 12 months of age. Table S4f. DPT-booster dose, delay if vaccinated after 24 months of age. Table S5. Western health Region EPI cluster survey. Proportion delayed to vaccination and predictors for delay by vaccine type using the adapted WHO recommended vaccine schedule. Table S5a. BCG, delay if vaccinated after 8 weeks of age. Table S5b. DPT1, delay if vaccinated after 3 months of age. Table S5c. DPT2, delay if vaccinated after 5 months of age. Table S5d. DPT3, delay if vaccinated after 7 months of age. Table S5e. Measles vaccine, delay if vaccinated after 12 months of age. Table S6. Greater Banjul health Clinics. Proportion delayed to vaccination and predictors for delay by vaccine type using the adapted WHO recommended vaccine schedule. Table S6a. BCG, delay if vaccinated after 8 weeks of age. Table S6b. DPT1, delay if vaccinated after 3 months of age. Table S6c. DPT2, delay if vaccinated after 5 months of age. Table S6d. DPT3, delay if vaccinated after 7 months of age. Table S6e. Measles vaccine, delay if vaccinated after 12 months of age.
(DOCX)
