# Supplementary material for: Coverage and Timing of Children's Vaccination: An Evaluation of the Expanded Programme on Immunisation in The Gambia
Source: PLoS One. 2014 Sep 18;9(9):e107280. doi: 10.1371/journal.pone.0107280 (PMC4169419; doi:10.1371/journal.pone.0107280)
Supplement: File S1 — This file contains Table S1–Table S6. Table S1. Estimated proportion vaccinated by age, vaccine type and geographical region in the Gambia. Table S1a. North bank region. Table S1b. South bank, Upper River Region. Table S1c. West Kiang, Lower River region. Table S1d. Western health region. Table S1e. Greater Banjul Health facilities, DPT booster dose not recorded. Table S2. North bank region: Proportion delayed to vaccination and predictors for delay by vaccine type using the adapted WHO recommended vaccine schedule. Table S2a. BCG, delay if vaccinated after 8 weeks of age. Table S2b. DPT1, delay if vaccinated after 3 months of age. Table S2c. DPT2, delay if vaccinated after 5 months of age. Table S2d. DPT3, delay if vaccinated after 7 months of age. Table S2e. Measles vaccine, delay if vaccinated after 12 months of age. Table S2f. DPT-booster dose, delay if vaccinated after 24 months of age. Table S3. South bank, upper river region. Proportion delayed to vaccination and predictors for delay by vaccine type using the adapted WHO recommended vaccine schedule. Table S3a. BCG, delay if vaccinated after 8 weeks of age. Table S3b. DPT1, delay if vaccinated after 3 months of age. Table S3c. DPT2, delay if vaccinated after 5 months of age. Table S3d. DPT3, delay if vaccinated after 7 months of age. Table S3e. Measles vaccine, delay if vaccinated after 12 months of age. Table S3f. DPT-booster dose, delay if vaccinated after 24 months of age. Table S4. West Kiang, Lower river region. Proportion delayed to vaccination and predictors for delay by vaccine type using the adapted WHO recommended vaccine schedule. Table S4a. BCG, delay if vaccinated after 8 weeks of age. Table S4b. DPT1, delay if vaccinated after 3 months of age. Table S4c. DPT2, delay if vaccinated after 5 months of age. Table S4d. DPT3, delay if vaccinated after 7 months of age. Table S4e. Measles vaccine, delay if vaccinated after 12 months of age. Table S4f. DPT-booster dose, delay if vaccinated after 24 months [file pone.0107280.s001.docx]

**Table S1: Estimated proportion vaccinated by age, vaccine type and geographical region in the Gambia**

**Table S1a: North bank region**

|  | BCG |  | DPT1 |  | DPT2 |  | DPT3 |  | Measles |  | DPT booster | |
| --- | --- | --- | --- | --- | --- | --- | --- | --- | --- | --- | --- | --- |
| *Age (mths)* | % | 95% CI | % | 95% CI | % | 95% CI | % | 95% CI | % | 95% CI | % | 95% CI |
| *1* | **61.28** | **(60.15,62.41)** |  |  |  |  |  |  |  |  |  |  |
| *2* | 87.48 | (86.7,88.24) | 13.89 | (13.1,14.72) |  |  |  |  |  |  |  |  |
| *3* | 91.12 | (90.44,91.77) | **69.71** | **(68.63,70.79)** | 8.16 | (7.53,8.83) |  |  |  |  |  |  |
| *4* | 92.33 | (91.69,92.93) | 86.49 | (85.67,87.29) | **49.73** | **(48.55,50.93)** | 5.44 | (4.92,6.02) |  |  |  |  |
| *5* | 92.75 | (92.13,93.34) | 90.9 | (90.2,91.57) | 74.7 | (73.65,75.74) | **34.14** | **(33,35.31)** |  |  |  |  |
| *6* | 93.28 | (92.68,93.85) | 92.5 | (91.85,93.11) | 84.35 | (83.46,85.22) | 57.48 | (56.27,58.7) |  |  |  |  |
| *7* | 93.49 | (92.9,94.06) | 93.1 | (92.47,93.69) | 88.36 | (87.56,89.13) | 70.39 | (69.25,71.52) |  |  |  |  |
| *8* | 93.65 | (93.06,94.21) | 93.61 | (93,94.18) | 89.95 | (89.2,90.68) | 76.74 | (75.68,77.79) |  |  |  |  |
| *9* | 93.77 | (93.18,94.32) | 93.82 | (93.21,94.38) | 90.75 | (90.02,91.45) | 80.16 | (79.14,81.15) | 17.45 | (16.47,18.49) |  |  |
| *10* | 93.85 | (93.27,94.4) | 93.98 | (93.38,94.54) | 91.26 | (90.54,91.94) | 82.57 | (81.6,83.53) | **62.43** | **(61.13,63.73)** |  |  |
| *12* | 94 | (93.42,94.54) | 94.21 | (93.62,94.76) | 91.88 | (91.18,92.55) | 84.51 | (83.57,85.43) | 84.13 | (83.11,85.12) |  |  |
| *13* | 94.7 | (94.14,95.22) | 94.33 | (93.75,94.88) | 92.14 | (91.44,92.8) | 85.09 | (84.15,86) | 86.73 | (85.77,87.66) |  |  |
| *15* | 94.94 | (94.39,95.45) | 94.82 | (94.25,95.36) | 92.48 | (91.79,93.13) | 85.86 | (84.93,86.76) | 88.58 | (87.66,89.46) |  |  |
| *18* | 95.03 | (94.48,95.54) | 95 | (94.43,95.53) | 93.18 | (92.51,93.82) | 87.34 | (86.42,88.23) | 89.85 | (88.96,90.7) | 29.01 | (27.27,30.84) |
| *19* | 95.08 | (94.54,95.59) | 95.11 | (94.54,95.64) | 93.27 | (92.6,93.91) | 87.65 | (86.73,88.54) | 90.17 | (89.29,91.01) | **37.82** | **(35.93,39.77)** |
| *24* | 95.23 | (94.67,95.74) | 95.36 | (94.79,95.89) | 93.63 | (92.95,94.27) | 88.18 | (87.25,89.07) | 93.29 | (92.45,94.08) | 58.61 | (56.47,60.75) |
| *25* | 95.23 | (94.67,95.74) | 95.41 | (94.84,95.94) | 93.68 | (93,94.31) | 88.24 | (87.3,89.13) | 93.34 | (92.49,94.13) | 59.99 | (57.83,62.16) |

**Table S1b: South bank, Upper River Region**

|  | BCG |  | DPT1 |  | DPT2 |  | DPT3 |  | Measles |  | DPT booster | |
| --- | --- | --- | --- | --- | --- | --- | --- | --- | --- | --- | --- | --- |
| *Age (mths)* | % | 95% CI | % | 95% CI | % | 95% CI | % | 95% CI | % | 95% CI | % | 95% CI |
| *1* | **58.97** | **(58.44,59.49)** |  |  |  |  |  |  |  |  |  |  |
| *2* | 83.32 | (82.92,83.72) | 10.77 | (10.45,11.11) |  |  |  |  |  |  |  |  |
| *3* | 89.16 | (88.83,89.49) | **55.84** | **(55.31,56.36)** | 5.52 | (5.28,5.77) |  |  |  |  |  |  |
| *4* | 91.04 | (90.73,91.34) | 77.4 | (76.95,77.84) | **34.41** | **(33.9,34.92)** | 3.21 | (3.03,3.41) |  |  |  |  |
| *5* | 91.75 | (91.45,92.04) | 85.2 | (84.82,85.57) | 58.68 | (58.15,59.2) | **20.26** | **(19.84,20.7)** |  |  |  |  |
| *6* | 92.39 | (92.1,92.67) | 88.68 | (88.34,89.02) | 72.5 | (72.02,72.98) | 39.43 | (38.91,39.96) |  |  |  |  |
| *7* | 92.77 | (92.49,93.04) | 90.51 | (90.19,90.82) | 79.38 | (78.94,79.81) | 53.52 | (52.99,54.06) |  |  |  |  |
| *8* | 93.04 | (92.76,93.3) | 91.57 | (91.26,91.86) | 83.25 | (82.85,83.65) | 62.69 | (62.17,63.22) |  |  |  |  |
| *9* | 93.23 | (92.96,93.49) | 92.24 | (91.95,92.52) | 85.51 | (85.13,85.89) | 68.21 | (67.7,68.71) | 13.68 | (13.31,14.07) |  |  |
| *10* | 93.42 | (93.15,93.68) | 92.66 | (92.38,92.93) | 86.89 | (86.53,87.26) | 71.96 | (71.47,72.45) | **46.06** | **(45.51,46.61)** |  |  |
| *12* | 93.78 | (93.52,94.04) | 93.26 | (92.99,93.53) | 88.58 | (88.23,88.92) | 76.56 | (76.09,77.02) | 72.23 | (71.72,72.73) |  |  |
| *13* | 94.33 | (94.08,94.58) | 93.53 | (93.26,93.79) | 89.07 | (88.73,89.41) | 77.79 | (77.33,78.25) | 76.42 | (75.94,76.9) |  |  |
| *15* | 94.72 | (94.47,94.95) | 93.97 | (93.71,94.22) | 89.83 | (89.5,90.16) | 79.56 | (79.11,80.01) | 80.37 | (79.92,80.82) |  |  |
| *18* | 94.89 | (94.65,95.12) | 94.49 | (94.24,94.74) | 90.79 | (90.47,91.11) | 81.97 | (81.54,82.4) | 82.93 | (82.49,83.36) | 15.84 | (15.39,16.3) |
| *19* | 94.92 | (94.68,95.15) | 94.58 | (94.33,94.82) | 91.01 | (90.69,91.32) | 82.69 | (82.27,83.12) | 83.42 | (82.99,83.85) | **22.64** | **(22.12,23.16)** |
| *24* | 95.08 | (94.84,95.31) | 94.9 | (94.65,95.14) | 91.52 | (91.21,91.83) | 84.42 | (84,84.83) | 85.83 | (85.41,86.24) | 43.09 | (42.45,43.74) |
| *25* | 95.21 | (94.97,95.44) | 94.93 | (94.69,95.17) | 91.62 | (91.3,91.92) | 84.54 | (84.13,84.96) | 86.05 | (85.64,86.46) | 44.54 | (43.89,45.19) |

**Table S1c West Kiang, Lower River region**

|  | BCG |  | DPT1 |  | DPT2 |  | DPT3 |  | Measles |  | DPT booster | |
| --- | --- | --- | --- | --- | --- | --- | --- | --- | --- | --- | --- | --- |
| *Age (mths)* | % | 95% CI | % | 95% CI | % | 95% CI | % | 95% CI | % | 95% CI | % | 95% CI |
| *1* | **51.1** | **(46.81,55.55)** | - | - | - | - | - | - | - | - | - | - |
| *2* | 99 | (97.79,99.62) | 41.99 | (37.85,46.39) | - | - | - | - | - | - | - | - |
| *3* | 99.8 | (98.93,99.98) | **96.08** | **(94.14,97.52)** | 39.41 | (35.32,43.8) | - | - | - | - | - | - |
| *4* | - | - | 98.63 | (97.3,99.38) | **90.39** | **(87.64,92.75)** | 32.81 | (28.9,37.08) | - | - | - | - |
| *5* | - | - | 99.61 | (98.67,99.92) | 94.9 | (92.75,96.58) | **65.81** | **(61.67,69.92)** | - | - | - | - |
| *6* | - | - | 99.61 | (98.67,99.92) | 98.63 | (97.3,99.38) | 86.52 | (83.38,89.34) | - | - | - | - |
| *7* | - | - | 99.61 | (98.67,99.92) | 98.86 | (97.58,99.53) | 90.2 | (87.39,92.61) | - | - | - | - |
| *8* | - | - | 99.61 | (98.67,99.92) | 98.86 | (97.58,99.53) | 90.82 | (88.08,93.15) | - | - | - | - |
| *9* | - | - | 99.61 | (98.67,99.92) | 98.86 | (97.58,99.53) | 93.54 | (91.13,95.49) | 45.64 | (41.32,50.2) | - | - |
| *10* | - | - | 99.61 | (98.67,99.92) | 98.86 | (97.58,99.53) | 94 | (91.65,95.88) | **73.03** | **(69.01,76.91)** | - | - |
| *12* | - | - | 99.61 | (98.67,99.92) | 98.86 | (97.58,99.53) | 94.77 | (92.49,96.53) | 91.5 | (88.8,93.78) | - | - |
| *13* | - | - | 99.61 | (98.67,99.92) | 99.14 | (97.9,99.72) | 95.06 | (92.81,96.78) | 92.58 | (90.01,94.7) | - | - |
| *15* | - | - | 99.61 | (98.67,99.92) | 99.14 | (97.9,99.72) | 95.37 | (93.14,97.04) | 93.25 | (90.76,95.27) | - | - |
| *18* | - | - | 99.61 | (98.67,99.92) | 99.14 | (97.9,99.72) | 95.98 | (93.81,97.56) | 93.25 | (90.76,95.27) | 49.77 | (45.18,54.55) |
| *19* | - | - | 99.61 | (98.67,99.92) | 99.14 | (97.9,99.72) | 96.35 | (94.19,97.87) | 93.25 | (90.76,95.27) | **72.34** | **(67.94,76.59)** |
| *24* | - | - | 99.61 | (98.67,99.92) | 99.14 | (97.9,99.72) | 97.16 | (95.02,98.54) | 94.57 | (91.96,96.55) | 87.12 | (83.13,90.56) |
| *25* | - | - | 99.61 | (98.67,99.92) | 99.14 | (97.9,99.72) | 97.16 | (95.02,98.54) | 94.57 | (91.96,96.55) | 91.41 | (87.53,94.47) |

**Table S1d: Western health region**

|  | BCG |  | DPT1 |  | DPT2 |  | DPT3 |  | Measles |  | DPT booster | |
| --- | --- | --- | --- | --- | --- | --- | --- | --- | --- | --- | --- | --- |
| *Age (mths)* | % | 95% CI | % | 95% CI | % | 95% CI | % | 95% CI | % | 95% CI | % | 95% CI |
| *1* | **80.61** | **(77.89,83.19)** | 1.08 | (0.59,2.01) | - | - | - | - | - | - | - | - |
| *2* | 92.13 | (90.19,93.81) | 12.15 | (10.2,14.43) | 0.22 | (0.05,0.86) | - | - | 0.11 | (0.02,0.77) | - | - |
| *3* | 94.71 | (93.07,96.08) | **65.84** | **(62.77,68.88)** | 4.77 | (3.57,6.35) | 0.33 | (0.1,1) | 0.11 | (0.02,0.77) | - | - |
| *4* | 96.12 | (94.67,97.27) | 85.36 | (82.99,87.55) | **38.68** | **(35.62,41.9)** | 3.03 | (2.1,4.36) | 0.11 | (0.02,0.77) | - | - |
| *5* | 96.24 | (94.81,97.37) | 92.08 | (90.22,93.71) | 68.8 | (65.79,71.76) | **21.67** | **(19.15,24.47)** | 0.22 | (0.05,0.86) | 0.21 | (0.03,1.45) |
| *6* | 96.47 | (95.08,97.57) | 94.58 | (92.98,95.91) | 81.69 | (79.13,84.11) | 45.52 | (42.37,48.79) | 0.33 | (0.11,1.01) | 0.41 | (0.1,1.64) |
| *7* | 96.83 | (95.49,97.86) | 95.66 | (94.21,96.84) | 87.54 | (85.32,89.57) | 62.34 | (59.22,65.47) | 0.33 | (0.11,1.01) | 0.41 | (0.1,1.64) |
| *8* | 96.83 | (95.49,97.86) | 96.1 | (94.7,97.21) | 90.47 | (88.46,92.25) | 73.63 | (70.75,76.43) | 0.65 | (0.29,1.44) | 0.62 | (0.2,1.91) |
| *9* | 96.94 | (95.63,97.95) | 96.75 | (95.45,97.75) | 93.39 | (91.67,94.87) | 79.38 | (76.71,81.93) | 8.35 | (6.74,10.33) | 0.62 | (0.2,1.91) |
| *10* | 97.06 | (95.76,98.05) | 96.85 | (95.58,97.84) | 94.15 | (92.51,95.54) | 84.26 | (81.83,86.53) | **54.27** | **(51.09,57.52)** | 1.03 | (0.43,2.46) |
| *12* | 97.06 | (95.76,98.05) | 96.96 | (95.71,97.93) | 94.91 | (93.36,96.2) | 87.54 | (85.31,89.58) | 84.06 | (81.62,86.35) | 2.89 | (1.72,4.83) |
| *13* | 97.66 | (96.47,98.53) | 97.29 | (96.09,98.2) | 95.02 | (93.48,96.29) | 89.33 | (87.22,91.22) | 88.04 | (85.83,90.06) | 3.71 | (2.35,5.83) |
| *15* | 97.78 | (96.62,98.62) | 97.51 | (96.35,98.38) | 95.38 | (93.89,96.61) | 89.96 | (87.9,91.81) | 91.28 | (89.26,93.06) | 4.54 | (3.01,6.81) |
| *18* | 97.78 | (96.62,98.62) | 97.76 | (96.64,98.58) | 96.42 | (95.01,97.53) | 91.26 | (89.24,93.04) | 93.56 | (91.66,95.15) | 20.21 | (16.9,24.06) |
| *19* | 97.78 | (96.62,98.62) | 97.76 | (96.64,98.58) | 96.42 | (95.01,97.53) | 91.97 | (89.92,93.74) | 93.56 | (91.66,95.15) | **30.01** | **(26.08,34.38)** |
| *24* | - | - | - | - | - | - | 92.26 | (90.19,94.04) | - | - | 55.81 | (49.63,62.2) |
| *25* | - | - | - | - | - | - | - | - | - | - | 55.81 | (49.63,62.2) |

**Table S1e: Greater Banjul Health facilities, DPT booster dose not recorded**

|  | BCG |  | DPT1 |  | DPT2 |  | DPT3 |  | Measles |  |
| --- | --- | --- | --- | --- | --- | --- | --- | --- | --- | --- |
| *Age (mths)* | % | 95% CI | % | 95% CI | % | 95% CI | % | 95% CI | % | 95% CI |
| *1* | **84.11** | **(82.04,86.06)** |  |  |  |  |  |  |  |  |
| *2* | 91.27 | (89.63,92.74) | 16.39 | (14.56,18.44) |  |  |  |  |  |  |
| *3* | 93.86 | (92.45,95.09) | **74.48** | **(72.18,76.74)** | 6.77 | (5.57,8.22) |  |  |  |  |
| *4* | 95.44 | (94.19,96.49) | 92.09 | (90.6,93.42) | **45.47** | **(42.91,48.12)** | 3.56 | (2.71,4.68) |  |  |
| *5* | 96.38 | (95.25,97.31) | 95.94 | (94.81,96.88) | 74.27 | (71.96,76.53) | **24.73** | **(22.56,27.08)** |  |  |
| *6* | 96.93 | (95.88,97.78) | 97.58 | (96.67,98.29) | 88.38 | (86.64,89.99) | 53.24 | (50.66,55.88) |  |  |
| *7* | 97.56 | (96.6,98.31) | 98.65 | (97.93,99.15) | 93.44 | (92.07,94.66) | 73.63 | (71.3,75.91) |  |  |
| *8* | 97.64 | (96.69,98.37) | 98.93 | (98.28,99.37) | 96.72 | (95.69,97.56) | 83.82 | (81.84,85.69) |  |  |
| *9* | 97.72 | (96.78,98.44) | 99.36 | (98.82,99.68) | 97.58 | (96.67,98.29) | 89.38 | (87.7,90.92) | 17.59 | (15.69,19.7) |
| *10* | 97.72 | (96.78,98.44) | 99.43 | (98.91,99.73) | 98.65 | (97.93,99.15) | 93.4 | (92.02,94.62) | **66.44** | **(63.94,68.93)** |
| *12* | 98.19 | (97.34,98.82) | 99.5 | (99.01,99.77) | 99.22 | (98.64,99.58) | 96.37 | (95.29,97.27) | 88.14 | (86.34,89.8) |
| *13* | 98.58 | (97.81,99.13) | 99.57 | (99.1,99.82) | 99.29 | (98.73,99.63) | 97.33 | (96.37,98.08) | 91.43 | (89.85,92.86) |
| *15* | 98.58 | (97.81,99.13) | 99.71 | (99.3,99.9) | 99.5 | (99.01,99.77) | 98.17 | (97.34,98.78) | 95.09 | (93.82,96.16) |
| *18* | 98.58 | (97.81,99.13) | 99.71 | (99.3,99.9) | 99.75 | (99.32,99.92) | 98.67 | (97.92,99.19) | 96.69 | (95.61,97.57) |
| *19* | 98.58 | (97.81,99.13) | 99.71 | (99.3,99.9) | 99.75 | (99.32,99.92) | 98.95 | (98.25,99.41) | 97.09 | (96.07,97.91) |
| *24* | 98.58 | (97.81,99.13) | 99.71 | (99.3,99.9) | 99.75 | (99.32,99.92) | 99.27 | (98.62,99.64) | 98.49 | (97.69,99.06) |
| *25* | 98.58 | (97.81,99.13) | 99.71 | (99.3,99.9) | 99.75 | (99.32,99.92) | 99.39 | (98.76,99.73) | 98.49 | (97.69,99.06) |

**Table S2: North bank region: Proportion delayed to vaccination and predictors for delay by vaccine type using the adapted WHO recommended vaccine schedule**

**Table S2a: BCG, delay if vaccinated after 8 weeks of age**

|  | Total vaccinated | % vaccinated who delay | Median delay from upper limit (wks) | IQR | Crude | 95% CI | Adjusted hazard ratio | 95% CI |
| --- | --- | --- | --- | --- | --- | --- | --- | --- |
| Overall | 6,738 | 9.26 | 3.71 | (1.14, 12.93) |  |  |  |  |
| Year of birth | |  |  |  |  |  |  |  |
| 2005 | 528 | 12.5 | 1.93 | (0.71, 5.86) | 1 |  | 1 |  |
| 2006 | 653 | 10.87 | 5.00 | (1.43, 10.86) | 0.96 | (0.85, 1.09) | 0.97 | (0.86, 1.10) |
| 2007 | 900 | 8.44 | 2.64 | (0.86, 9.07) | 0.87 | (0.77, 0.97) | 0.87 | (0.77, 0.97) |
| 2008 | 728 | 11.26 | 5.00 | (1.71, 15.57) | 0.97 | (0.86, 1.09) | 0.96 | (0.86, 1.09) |
| 2009 | 1251 | 10.31 | 6.00 | (1.29, 29.14) | 0.89 | (0.79, 0.99) | 0.89 | (0.79, 0.99) |
| 2010 | 1414 | 8.35 | 2.64 | (1.14, 9.00) | 0.83 | (0.74, 0.92) | 0.83 | (0.74, 0.92) |
| 2011 | 1264 | 6.49 | 3.00 | (1.00, 8.86) | 0.81 | (0.73, 0.90) | 0.82 | (0.73, 0.91) |
| Ethnic group | |  |  |  |  |  |  |  |
| Fula | 1,445 | 12.6 | 3.43 | (1.00, 8.14) | 1 |  | 1 |  |
| Mandinka | 1,968 | 6.96 | 2.86 | (1.14, 10.14) | 0.76 | (0.70, 0.81) | 0.76 | (0.70, 0.81) |
| Wollof | 3,074 | 9.11 | 4.64 | (1.29, 15.00) | 0.82 | (0.77, 0.87) | 0.82 | (0.77, 0.88) |
| Others | 251 | 9.96 | 5.00 | (1.00, 11.43) | 0.97 | (0.84, 1.11) | 0.97 | (0.84, 1.12) |
| Gender |  |  |  |  |  |  |  |  |
| Male | 3,415 | 9.43 | 3.71 | (1.14, 10.43) | 1 |  |  |  |
| Female | 3,323 | 9.09 | 3.71 | (1.14, 14.43) | 0.98 | (0.93, 1.03) |  |  |

Adjusted only by other variables which were significant in unadjusted analysis

**Table S2b: DPT1, delay if vaccinated after** 3 months of age

|  | Total vaccinated | % vaccinated who delay | Median delay from upper limit (wks) | IQR | Crude | 95% CI | Adjusted hazard ratio | 95% CI |
| --- | --- | --- | --- | --- | --- | --- | --- | --- |
| Overall | 6,538 | 8.61 | 4.00 | (1.57, 10.71) |  |  |  |  |
| Year of birth | |  |  |  |  |  |  |  |
| 2005 | 549 | 9.84 | 4.43 | (1.71, 12.86) | 1 |  | 1 |  |
| 2006 | 665 | 10.38 | 3.71 | (1.71, 12.29) | 0.96 | (0.85, 1.08) | 0.97 | (0.86, 1.09) |
| 2007 | 903 | 8.64 | 2.86 | (1.42, 7.43) | 0.85 | (0.76, 0.95) | 0.87 | (0.78, 0.98) |
| 2008 | 683 | 14.06 | 4.43 | (1.50, 21.71) | 1.04 | (0.92, 1.17) | 1.02 | (0.90, 1.15) |
| 2009 | 1265 | 9.72 | 5.14 | (1.71, 13.57) | 0.89 | (0.80, 0.98) | 0.85 | (0.77, 0.95) |
| 2010 | 1371 | 6.42 | 3.36 | (1.29, 7.57) | 0.76 | (0.68, 0.84) | 0.75 | (0.67, 0.83) |
| 2011 | 1102 | 4.99 | 3.29 | (1.57, 5.86) | 0.72 | (0.65, 0.80) | 0.74 | (0.66, 0.82) |
| Ethnic group | |  |  |  |  |  |  |  |
| Fula | 1,404 | 11.04 | 4.14 | (1.71, 9.29) | 1 |  | 1 |  |
| Mandinka | 1,927 | 6.69 | 3.29 | (1.14, 10.14) | 0.83 | (0.77, 0.89) | 0.88 | (0.82, 0.95) |
| Wollof | 2,965 | 8.67 | 4.00 | (1.57, 12.00) | 0.89 | (0.84, 0.96) | 0.93 | (0.87, 1.00) |
| Others | 242 | 9.09 | 3.29 | (1.29, 7.71) | 0.89 | (0.77, 1.03) | 0.91 | (0.79, 1.05) |
| Gender |  |  |  |  |  |  |  |  |
| Male | 3,315 | 8.84 | 3.71 | (1.57, 9.43) | 1 |  |  |  |
| Female | 3,223 | 8.38 | 4.14 | (1.57, 12.00) | 1.01 | (0.96, 1.06) |  |  |
| BCG vaccination delayed | |  |  |  |  |  |  |  |
| No | 5,738 | 5.07 | 2.71 | (1.14, 5.86) | 1 |  | 1 |  |
| Yes | 572 | 42.83 | 5.57 | (2.14, 14.86) | 3.84 | (3.43, 4.30) | 3.78 | (3.38, 4.24) |

Adjusted only by other variables which were significant in unadjusted analysis

**Table S2c: DPT2 , delay if vaccinated after** 5 months

|  | Total vaccinated | % vaccinated who delay | Median delay from upper limit (wks) | IQR | Crude | 95% CI | Adjusted hazard ratio | 95% CI |
| --- | --- | --- | --- | --- | --- | --- | --- | --- |
| Overall | 6,163 | 8.71 | 4.86 | (1.71, 13.86) |  |  |  |  |
| Year of birth | |  |  |  |  |  |  |  |
| 2005 | 537 | 10.61 | 6.00 | (1.86, 13.86) | 1 |  | 1 |  |
| 2006 | 645 | 13.02 | 3.71 | (1.43, 11.29) | 0.99 | (0.88, 1.12) | 1.00 | (0.89, 1.13) |
| 2007 | 860 | 9.42 | 3.86 | (1.57, 11.00) | 0.85 | (0.76, 0.95) | 0.88 | (0.78, 0.98) |
| 2008 | 633 | 14.06 | 6.86 | (2.71, 31.71) | 1.05 | (0.93, 1.19) | 0.99 | (0.88, 1.12) |
| 2009 | 1215 | 9.71 | 5.57 | (2.29, 16.29) | 0.85 | (0.77, 0.95) | 0.86 | (0.78, 0.96) |
| 2010 | 1300 | 5.46 | 4.42 | (1.71, 15.43) | 0.66 | (0.60, 0.74) | 0.67 | (0.60, 0.74) |
| 2011 | 973 | 3.8 | 3.85 | (1.43, 8.86) | 0.60 | (0.54, 0.67) | 0.64 | (0.57, 0.71) |
| Ethnic group | |  |  |  |  |  |  |  |
| Fula | 1,332 | 10.96 | 5.21 | (2.42, 16.14) | 1 |  | 1 |  |
| Mandinka | 1,812 | 7.34 | 3.57 | (0.86, 11.71) | 0.82 | (0.76, 0.88) | 0.88 | (0.82, 0.95) |
| Wollof | 2,796 | 8.4 | 5.00 | (1.86, 13.29) | 0.90 | (0.84, 0.96) | 0.96 | (0.90, 1.03) |
| Others | 223 | 10.31 | 5.57 | (2.71, 16.43) | 0.95 | (0.82, 1.11) | 0.98 | (0.84, 1.14) |
| Gender |  |  |  |  |  |  |  |  |
| Male | 3,121 | 8.27 | 4.86 | (1.86, 15.43) | 1 |  | - |  |
| Female | 3,042 | 9.17 | 4.86 | (1.57, 13.86) | 1.02 | (0.97, 1.07) |  |  |
| No. previous of delayed vaccinations | | |  |  |  |  |  |  |
| 0 | 5,364 | 3.58 | 3.57 | (1.43, 8.93) | 1 |  | 1 |  |
| 1 | 574 | 31.53 | 4.29 | (1.29, 12.71) | 3.43 | (3.09, 3.80) | 3.33 | (3.00, 3.70) |
| 2 | 225 | 72.89 | 8.64 | (3.43, 22.79) | 12.21 | (9.47, 15.74) | 11.96 | (9.27, 15.41) |

Adjusted only by other variables which were significant in unadjusted analysis

**Table S2d: DPT3, delay if vaccinated after** 7 months **of age**

|  | Total vaccinated | % vaccinated who delay | Median delay from upper limit (wks) | IQR | Crude | 95% CI | Adjusted hazard ratio | 95% CI |
| --- | --- | --- | --- | --- | --- | --- | --- | --- |
| Overall | 5,418 | 10.5 | 6.71 | (2.57, 16.86) |  |  |  |  |
| Year of birth | |  |  |  |  |  |  |  |
| 2005 | 514 | 16.93 | 6.71 | (3.29, 16.00) | 1 |  | 1 |  |
| 2006 | 607 | 14.83 | 6.64 | (2.00, 15.86) | 0.90 | (0.79, 1.02) | 0.86 | (0.76, 0.98) |
| 2007 | 764 | 10.73 | 6.43 | (3.43, 14.71) | 0.74 | (0.66, 0.84) | 0.74 | (0.66, 0.84) |
| 2008 | 535 | 17.2 | 10.14 | (4.14, 27.29) | 0.92 | (0.80, 1.05) | 0.86 | (0.75, 0.98) |
| 2009 | 1069 | 10.48 | 6.36 | (1.79, 16.00) | 0.70 | (0.62, 0.78) | 0.69 | (0.62, 0.77) |
| 2010 | 1153 | 6.76 | 6.07 | (2.43, 14.57) | 0.55 | (0.49, 0.61) | 0.54 | (0.49, 0.61) |
| 2011 | 776 | 3.61 | 5.21 | (1.50, 8.64) | 0.46 | (0.41, 0.52) | 0.47 | (0.41, 0.52) |
| Ethnic group | |  |  |  |  |  |  |  |
| Fula | 1,167 | 12.17 | 6.14 | (2.86, 17.14) | 1 |  | 1 |  |
| Mandinka | 1,596 | 8.96 | 7.57 | (3.43, 22.29) | 0.82 | (0.76, 0.89) | 0.86 | (0.79, 0.93) |
| Wollof | 2,462 | 10.56 | 6.79 | (2.43, 15.29) | 0.94 | (0.87, 1.01) | 1.01 | (0.94, 1.09) |
| Others | 193 | 12.44 | 6.00 | (1.64, 14.14) | 0.97 | (0.82, 1.14) | 1.00 | (0.85, 1.17) |
| Gender |  |  |  |  |  |  |  |  |
| Male | 2,755 | 9.87 | 7.29 | (2.86, 20.50) | 1 |  |  |  |
| Female | 2,663 | 11.15 | 6.14 | (2.57, 14.29) | 1.04 | (0.98, 1.10) |  |  |
| No. previous of delayed vaccinations | | |  |  |  |  |  |  |
| 0 | 4,634 | 5.16 | 5.57 | (1.71, 13.71) | 1 |  | 1 |  |
| 1 | 479 | 29.23 | 6.14 | (3.21, 14.14) | 2.89 | (2.59, 3.23) | 2.85 | (2.55, 3.19) |
| 2 | 177 | 50.85 | 8.21 | (2.43, 21.57) | 5.63 | (4.55, 6.97) | 5.45 | (4.40, 6.74) |
| 3 | 128 | 78.13 | 11.50 | (6.07, 27.14) | 14.00 | (9.65, 20.32) | 13.74 | (9.46, 19.94) |

Adjusted only by other variables which were significant in unadjusted analysis

**Table S2e: Measles vaccine, delay if vaccinated after 12 months of age**

|  | Total vaccinated | % vaccinated who delay | Median delay from upper limit (wks) | IQR | Crude | 95% CI | Adjusted hazard ratio | 95% CI |
| --- | --- | --- | --- | --- | --- | --- | --- | --- |
| Overall | 4,791 | 4.97 | 18.43 | (4.93, 35.93) |  |  |  |  |
| Year of birth | |  |  |  |  |  |  |  |
| 2005 | 485 | 7.01 | 21.50 | (4.79, 37.64) | 1 |  | 1 |  |
| 2006 | 558 | 7.17 | 14.00 | (3.00, 25.93) | 0.95 | (0.84, 1.08) | 0.94 | (0.83, 1.07) |
| 2007 | 623 | 3.05 | 30.93 | (3.36, 39.64) | 0.81 | (0.72, 0.92) | 0.83 | (0.73, 0.93) |
| 2008 | 588 | 8.67 | 27.21 | (7.79, 35.79) | 0.94 | (0.83, 1.06) | 0.93 | (0.82, 1.05) |
| 2009 | 1118 | 4.65 | 19.64 | (6.07, 36.43) | 0.83 | (0.75, 0.93) | 0.86 | (0.77, 0.96) |
| 2010 | 1002 | 3.79 | 11.07 | (4.21, 35.07) | 0.75 | (0.67, 0.84) | 0.78 | (0.70, 0.88) |
| 2011 | 417 | 0.96 | 10.29 | (9.00, 12.14) | 0.58 | (0.51, 0.67) | 0.63 | (0.55, 0.72) |
| Ethnic group | |  |  |  |  |  |  |  |
| Fula | 1,073 | 5.13 | 18.93 | (5.21, 36.64) | 1 |  |  |  |
| Mandinka | 1,344 | 4.46 | 27.57 | (8.64, 38.29) | 0.94 | (0.87, 1.02) |  |  |
| Wollof | 2,211 | 5.29 | 13.79 | (4.50, 34.64) | 1.00 | (0.93, 1.08) |  |  |
| Others | 163 | 3.68 | 5.64 | (3.79, 11.50) | 1.00 | (0.84, 1.18) |  |  |
| Gender |  |  |  |  |  |  |  |  |
| Male | 2,433 | 5.22 | 19.21 | (5.64, 35.07) | 1 |  |  |  |
| Female | 2,358 | 4.71 | 13.07 | (4.50, 36.50) | 1.02 | (0.96, 1.08) |  |  |
| No. previous of delayed vaccinations | | |  |  |  |  |  |  |
| 0 | 3,743 | 3.15 | 20.21 | (4.93, 36.50) | 1 |  | 1 |  |
| 1 | 597 | 6.37 | 12.79 | (2.36, 34.64) | 1.30 | (1.19, 1.43) | 1.27 | (1.16, 1.39) |
| 2 | 246 | 10.98 | 15.07 | (3.79, 34.36) | 1.68 | (1.46, 1.93) | 1.62 | (1.41, 1.86) |
| 3 | 114 | 14.04 | 11.29 | (6.14, 32.14) | 2.04 | (1.67, 2.49) | 1.94 | (1.59, 2.38) |
| 4 | 91 | 42.86 | 19.21 | (6.36, 36.64) | 4.47 | (3.39, 5.88) | 4.36 | (3.31, 5.73) |
|  |  |  |  |  |  |  |  |  |

Adjusted only by other variables which were significant in unadjusted analysis

**Table S2f: DPT-booster dose, delay if vaccinated after 2**4 months **of age**

|  | Total vaccinated | % vaccinated who delay | Median delay from upper limit (wks) | IQR | Crude | 95% CI | Adjusted hazard ratio | 95% CI |
| --- | --- | --- | --- | --- | --- | --- | --- | --- |
| Overall | 1,238 | 3.63 | 11.79 | (4.93, 23.50) |  |  |  |  |
| Year of birth | |  |  |  |  |  |  |  |
| 2005 | 275 | 7.27 | 9.50 | (3.57, 16.71) | 1 |  | 1 |  |
| 2006 | 236 | 2.97 | 27.93 | (7.36, 45.50) | 0.69 | (0.58, 0.83) | 0.63 | (0.53, 0.75) |
| 2007 | 185 | 4.86 | 13.93 | (4.93, 25.93) | 0.78 | (0.65, 0.95) | 0.75 | (0.62, 0.91) |
| 2008 | 287 | 2.09 | 11.64 | (10.21, 13.79) | 0.58 | (0.49, 0.68) | 0.55 | (0.46, 0.65) |
| 2009 | 255 | 1.18 | 14.93 | (4.07, 28.50) | 0.46 | (0.38, 0.55) | 0.47 | (0.39, 0.56) |
| Ethnic group | |  |  |  |  |  |  |  |
| Fula | 284 | 3.87 | 13.07 | (4.50, 23.60) | 1 |  |  |  |
| Mandinka | 400 | 5 | 9.29 | (4.21, 26.00) | 0.98 | (0.84, 1.15) |  |  |
| Wollof | 519 | 2.5 | 11.79 | (8.93, 23.36) | 0.95 | (0.82, 1.10) |  |  |
| Others | 35 | 2.86 | 14.50 | (0.00, 0.00) | 1.00 | (0.70, 1.43) |  |  |
| Gender |  |  |  |  |  |  |  |  |
| Male | 644 | 4.04 | 14.14 | (4.36, 25.93) | 1 |  |  |  |
| Female | 594 | 3.2 | 11.50 | (4.93, 14.93) | 0.92 | (0.82, 1.03) |  |  |
| No. previous of delayed vaccinations | | |  |  |  |  |  |  |
| 0 | 950 | 2.42 | 11.79 | (3.50, 23.36) | 1 |  | 1 |  |
| 1 | 175 | 5.14 | 13.92 | (9.20, 27.93) | 1.35 | (1.15, 1.60) | 1.35 | (1.14, 1.60) |
| 2 | 59 | 3.39 | 15.43 | (7.36, 23.50) | 1.73 | (1.32, 2.26) | 1.77 | (1.35, 2.31) |
| 3 | 34 | 14.71 | 4.93 | (4.36, 13.76) | 2.40 | (1.66, 3.48) | 2.43 | (1.67, 3.52) |
| 4 | 12 | 16.67 | 24.07 | (10.36, 37.79) | 2.98 | (1.60, 5.57) | 2.74 | (1.47, 5.13) |
| 5 | 8 | 50 | 10.79 | (8.64, 67.00) | 6.22 | (2.33, 16.64) | 6.76 | (2.52, 18.12) |

*Exclude 2010 and 2011 as follow up for data collected less than WHO recommended age range.* Adjusted only by other variables which were significant in unadjusted analysis

**Table S3: South bank, upper river region: Proportion delayed to vaccination and predictors for delay by vaccine type using the adapted WHO recommended vaccine schedule**

**Table S3a: BCG, delay if vaccinated after 8 weeks of age**

|  | Total vaccinated | % vaccinated who delay | Median delay from upper limit (wks) | IQR | Crude | 95% CI | Adjusted hazard ratio | 95% CI |
| --- | --- | --- | --- | --- | --- | --- | --- | --- |
| Overall | 32,490 | 14.6 | 4.14 | (1.43, 14.86) |  |  |  |  |
| Year of birth | |  |  |  |  |  |  |  |
| 2005 | 1979 | 20.31 | 6.07 | (1.86, 36.71) | 1 |  | 1 |  |
| 2006 | 3320 | 19.07 | 4.71 | (1.57, 22.43) | 0.98 | (0.92, 1.04) | 1.00 | (0.94, 1.06) |
| 2007 | 4531 | 18.67 | 5.00 | (1.86, 20.14) | 0.93 | (0.88, 0.99) | 0.95 | (0.89, 1.01) |
| 2008 | 5416 | 16.65 | 3.93 | (1.43, 12.86) | 0.92 | (0.87, 0.97) | 0.93 | (0.88, 0.98) |
| 2009 | 5733 | 11.06 | 3.71 | (1.29, 10.86) | 0.75 | (0.71, 0.79) | 0.75 | (0.71, 0.80) |
| 2010 | 5,738 | 12.29 | 5.00 | (1.57, 16.43) | 0.77 | (0.72, 0.81) | 0.77 | (0.73, 0.81) |
| 2011 | 5,773 | 10.74 | 2.71 | (1.00, 6.39) | 0.75 | (0.71, 0.79) | 0.74 | (0.70, 0.78) |
| Ethnic group | |  |  |  |  |  |  |  |
| Fula | 10,022 | 19.46 | 3.86 | (1.43, 11.43) | 1 |  | 1 |  |
| Mandinka | 6,915 | 13.42 | 3.14 | (1.29, 11.14) | 0.78 | (0.76, 0.81) | 0.77 | (0.75, 0.80) |
| Wollof | 244 | 15.16 | 3.43 | (1.14, 13.57) | 0.93 | (0.81, 1.07) | 0.94 | (0.82, 1.08) |
| Serrehule | 14,744 | 11.79 | 5.14 | (1.71, 21.71) | 0.67 | (0.65, 0.68) | 0.66 | (0.64, 0.68) |
| Other | 462 | 14.94 | 4.29 | (1.86, 17.43) | 0.82 | (0.74, 0.90) | 0.80 | (0.73, 0.89) |
| Gender |  |  |  |  |  |  |  |  |
| Male | 16,614 | 14.44 | 4.14 | (1.43, 14.57) | 1 |  |  |  |
| Female | 15,870 | 14.76 | 4.14 | (1.43, 15.43) | 1.01 | (0.99, 1.04) |  |  |

Adjusted only by other variables which were significant in unadjusted analysis

**Table S3b: DPT1, delay if vaccinated after** 3 months **of age**

|  | Total vaccinated | % vaccinated who delay | Median delay from upper limit (wks) | IQR | Crude | 95% CI | Adjusted hazard ratio | 95% CI |
| --- | --- | --- | --- | --- | --- | --- | --- | --- |
| Overall | 32,311 | 18.83 | 5.00 | (1.86, 13.00) |  |  |  |  |
| Year of birth | |  |  |  |  |  |  |  |
| 2005 | 1998 | 23.07 | 6.29 | (2.00, 26.71) | 1 |  | 1 |  |
| 2006 | 3326 | 23.54 | 5.71 | (1.86, 15.14) | 1.06 | (0.99, 1.12) | 1.06 | (0.99, 1.13) |
| 2007 | 4714 | 24.46 | 6.14 | (2.29, 16.71) | 1.08 | (1.02, 1.15) | 1.10 | (1.03, 1.17) |
| 2008 | 5560 | 23.65 | 5.00 | (1.86, 13.43) | 1.08 | (1.02, 1.15) | 1.13 | (1.06, 1.20) |
| 2009 | 5418 | 15.17 | 4.43 | (1.71, 10.29) | 0.86 | (0.81, 0.91) | 0.93 | (0.88, 0.99) |
| 2010 | 5,715 | 14.68 | 4.43 | (1.71, 11.29) | 0.84 | (0.80, 0.89) | 0.90 | (0.85, 0.96) |
| 2011 | 5,580 | 12.74 | 3.86 | (1.57, 8.29) | 0.79 | (0.75, 0.84) | 0.86 | (0.81, 0.91) |
| Ethnic group | |  |  |  |  |  |  |  |
| Fula | 9,908 | 21.91 | 5.29 | (2.00, 13.57) | 1 |  | 1 |  |
| Mandinka | 6,869 | 17.24 | 4.14 | (1.71, 10.36) | 0.86 | (0.83, 0.89) | 0.91 | (0.88, 0.94) |
| Wollof | 241 | 20.33 | 4.86 | (1.86, 10.71) | 0.94 | (0.81, 1.08) | 0.97 | (0.84, 1.12) |
| Serrehule | 14,734 | 17.52 | 5.14 | (1.86, 13.57) | 0.85 | (0.82, 0.87) | 0.91 | (0.89, 0.94) |
| Other | 456 | 16.89 | 6.29 | (2.86, 15.71) | 0.84 | (0.75, 0.93) | 0.86 | (0.77, 0.96) |
| Gender |  |  |  |  |  |  |  |  |
| Male | 16,540 | 18.68 | 4.86 | (1.86, 12.86) | 1 |  |  |  |
| Female | 15,765 | 18.99 | 5.00 | (1.86, 13.43) | 1.01 | (0.99, 1.04) |  |  |
| Delayed BCG vaccination | |  |  |  |  |  |  |  |
| No | 26,634 | 12.23 | 3.43 | (1.43, 8.29) | 1 |  | 1 |  |
| Yes | 4,437 | 56.07 | 7.57 | (2.71, 20.90) | 3.80 | (3.62, 3.98) | 3.69 | (3.52, 3.87) |

Adjusted only by other variables which were significant in unadjusted analysis

**Table S3c: DPT2, delay if vaccinated after** 5 months **of age**

|  | Total vaccinated | % vaccinated who delay | Median delay from upper limit (wks) | IQR | Crude | 95% CI | Adjusted hazard ratio | 95% CI |
| --- | --- | --- | --- | --- | --- | --- | --- | --- |
| Overall | 30,614 | 20.56 | 6.71 | (2.57, 16.71) |  |  |  |  |
| Year of birth | |  |  |  |  |  |  |  |
| 2005 | 1955 | 25.32 | 10.14 | (3.43, 35.86) | 1 |  | 1 |  |
| 2006 | 3234 | 25.08 | 7.86 | (2.86, 19.71) | 1.05 | (0.99, 1.12) | 1.10 | (1.03, 1.18) |
| 2007 | 4568 | 27.61 | 7.86 | (3.14, 20.57) | 1.12 | (1.05, 1.19) | 1.19 | (1.12, 1.27) |
| 2008 | 5328 | 24.31 | 7.14 | (2.71, 16.57) | 1.02 | (0.96, 1.08) | 1.09 | (1.03, 1.16) |
| 2009 | 4967 | 16.13 | 5.86 | (2.29, 15.29) | 0.77 | (0.73, 0.82) | 0.90 | (0.85, 0.95) |
| 2010 | 5,498 | 17.5 | 6.00 | (2.29, 13.00) | 0.83 | (0.79, 0.88) | 1.01 | (0.95, 1.07) |
| 2011 | 5,064 | 13.21 | 4.43 | (2.00, 9.43) | 0.71 | (0.67, 0.76) | 0.89 | (0.83, 0.94) |
| Ethnic group | |  |  |  |  |  |  |  |
| Fula | 9,335 | 23.94 | 6.57 | (2.71, 16.86) | 1 |  | 1 |  |
| Mandinka | 6,556 | 18.21 | 6.00 | (2.43, 14.86) | 0.84 | (0.81, 0.87) | 0.91 | (0.88, 0.94) |
| Wollof | 225 | 16.89 | 6.50 | (1.71, 11.71) | 0.81 | (0.70, 0.94) | 0.81 | (0.70, 0.94) |
| Serrehule | 13,978 | 19.52 | 7.14 | (2.71, 17.29) | 0.86 | (0.83, 0.88) | 0.93 | (0.91, 0.96) |
| Other | 425 | 19.53 | 7.57 | (2.86, 19.29) | 0.86 | (0.77, 0.96) | 0.91 | (0.81, 1.01) |
| Gender |  |  |  |  |  |  |  |  |
| Male | 15,699 | 20.21 | 6.71 | (2.57, 16.57) | 1 |  |  |  |
| Female | 14,910 | 20.93 | 6.86 | (2.57, 16.76) | 1.02 | (1.00, 1.05) |  |  |
| No. previous of delayed vaccinations | | |  |  |  |  |  |  |
| 0 | 23,352 | 7.99 | 3.71 | (1.57, 8.29) | 1 |  | 1 |  |
| 1 | 4,990 | 51.02 | 7.43 | (2.86, 16.43) | 4.10 | (3.93, 4.28) | 4.04 | (3.87, 4.22) |
| 2 | 2,272 | 82.83 | 11.00 | (4.43, 29.14) | 14.66 | (13.25, 16.21) | 14.18 | (12.82, 15.69) |

Adjusted only by other variables which were significant in unadjusted analysis

**Table S3d: DPT3, delay if vaccinated after** 7 months **of age**

|  | Total vaccinated | % vaccinated who delay | Median delay from upper limit (wks) | IQR | Crude | 95% CI | Adjusted hazard ratio | 95% CI |
| --- | --- | --- | --- | --- | --- | --- | --- | --- |
| Overall | 27,554 | 24.78 | 10.42 | (4.00, 26.71) | |  |  |  |
| Year of birth | |  |  |  |  |  |  |  |
| 2005 | 1817 | 30.71 | 17.71 | (5.71, 47.86) | 1 |  | 1 |  |
| 2006 | 3028 | 31.8 | 13.71 | (4.43, 39.43) | 1.07 | (1.00, 1.15) | 1.12 | (1.05, 1.21) |
| 2007 | 4299 | 35.52 | 13.00 | (4.71, 30.43) | 1.16 | (1.09, 1.24) | 1.22 | (1.14, 1.30) |
| 2008 | 4831 | 27.8 | 9.86 | (4.00, 23.00) | 0.94 | (0.89, 1.01) | 1.01 | (0.94, 1.07) |
| 2009 | 4294 | 18.63 | 9.86 | (3.64, 29.93) | 0.69 | (0.65, 0.74) | 0.80 | (0.75, 0.85) |
| 2010 | 5,075 | 21.81 | 8.00 | (3.42, 17.00) | 0.79 | (0.74, 0.84) | 0.95 | (0.89, 1.01) |
| 2011 | 4,210 | 12.61 | 5.86 | (2.29, 12.29) | 0.58 | (0.55, 0.62) | 0.73 | (0.69, 0.78) |
| Ethnic group | |  |  |  |  |  |  |  |
| Fula | 8,305 | 26.88 | 10.21 | (3.86, 24.57) | 1 |  | 1 |  |
| Mandinka | 5,943 | 22.53 | 10.57 | (3.86, 27.26) | 0.86 | (0.83, 0.89) | 0.94 | (0.90, 0.97) |
| Wollof | 198 | 21.21 | 10.76 | (5.71, 31.29) | 0.85 | (0.72, 0.99) | 0.87 | (0.74, 1.02) |
| Serrehule | 12,659 | 24.39 | 10.57 | (4.14, 28.21) | 0.90 | (0.87, 0.93) | 0.98 | (0.95, 1.01) |
| Other | 365 | 28.49 | 11.14 | (3.57, 29.07) | 0.97 | (0.86, 1.10) | 1.05 | (0.92, 1.18) |
| Gender |  |  |  |  |  |  |  |  |
| Male | 14,198 | 24.83 | 10.57 | (4.00, 26.57) | 1 |  |  |  |
| Female | 13,353 | 24.74 | 10.43 | (4.00, 27.00) | 1.00 | (0.97, 1.03) |  |  |
| No. previous of delayed vaccinations | | |  |  |  |  |  |  |
| 0 | 20,018 | 10.67 | 7.43 | (2.71, 20.57) | 1 |  | 1 |  |
| 1 | 3,697 | 42.47 | 8.43 | (3.29, 19.57) | 2.92 | (2.79, 3.06) | 2.88 | (2.75, 3.01) |
| 2 | 2,278 | 75.86 | 12.57 | (5.29, 27.86) | 9.11 | (8.37, 9.92) | 8.82 | (8.10, 9.61) |
| 3 | 1,561 | 89.43 | 17.14 | (7.07, 37.64) | 21.90 | (18.78, 25.53) | 20.77 | (17.81, 24.22) |

**Table S3e: Measles vaccine, delay if vaccinated after** 12 months **of age**

|  | Total vaccinated | % vaccinated who delay | Median delay from upper limit (wks) | IQR | Crude | 95% CI | Adjusted hazard ratio | 95% CI |
| --- | --- | --- | --- | --- | --- | --- | --- | --- |
| Overall | 26,387 | 10.5 | 12.50 | (4.64, 33.93) |  |  |  |  |
| Year of birth | |  |  |  |  |  |  |  |
| 2005 | 1935 | 15.97 | 25.07 | (8.21, 44.64) | 1 |  | 1 |  |
| 2006 | 3216 | 14.37 | 15.14 | (5.79, 38.21) | 1.00 | (0.94, 1.06) | 1.01 | (0.95, 1.07) |
| 2007 | 4537 | 15.47 | 13.36 | (5.07, 34.07) | 1.07 | (1.01, 1.13) | 1.07 | (1.01, 1.14) |
| 2008 | 4769 | 10.34 | 10.79 | (3.93, 28.36) | 0.88 | (0.83, 0.93) | 0.89 | (0.84, 0.95) |
| 2009 | 3956 | 8.52 | 15.36 | (4.92, 35.76) | 0.84 | (0.79, 0.89) | 0.93 | (0.88, 0.99) |
| 2010 | 4,905 | 7.62 | 8.21 | (3.64, 17.07) | 0.81 | (0.76, 0.85) | 0.88 | (0.83, 0.93) |
| 2011 | 3,069 | 3.06 | 5.64 | (2.79, 10.64) | 0.64 | (0.60, 0.68) | 0.71 | (0.67, 0.76) |
| Ethnic group | |  |  |  |  |  |  |  |
| Fula | 7,977 | 11.31 | 11.93 | (4.64, 31.64) | 1 |  | 1 |  |
| Mandinka | 5,678 | 9.16 | 12.64 | (4.50, 35.36) | 0.91 | (0.88, 0.94) | 0.96 | (0.92, 0.99) |
| Wollof | 190 | 8.95 | 10.36 | (3.64, 30.21) | 0.96 | (0.83, 1.12) | 1.01 | (0.87, 1.17) |
| Serrehule | 12,110 | 10.61 | 12.93 | (4.93, 33.93) | 0.94 | (0.92, 0.97) | 0.98 | (0.96, 1.01) |
| Other | 350 | 10.57 | 11.79 | (6.79, 33.21) | 0.92 | (0.82, 1.03) | 0.92 | (0.82, 1.03) |
| Gender |  |  |  |  |  |  |  |  |
| Male | 13,576 | 10.42 | 12.21 | (4.79, 33.93) | 1 |  |  |  |
| Female | 12,807 | 10.6 | 12.64 | (4.64, 33.50) | 1.02 | (0.99, 1.04) |  |  |
| No. previous of delayed vaccinations | | |  |  |  |  |  |  |
| 0 | 16,372 | 5.17 | 11.64 | (4.36, 32.50) | 1 |  | 1 |  |
| 1 | 4,390 | 10.62 | 10.21 | (3.64, 30.36) | 1.37 | (1.32, 1.42) | 1.34 | (1.30, 1.39) |
| 2 | 2,435 | 15.11 | 10.36 | (4.50, 22.57) | 1.68 | (1.61, 1.76) | 1.65 | (1.57, 1.73) |
| 3 | 1,936 | 23.76 | 11.07 | (4.00, 29.07) | 2.26 | (2.14, 2.38) | 2.19 | (2.07, 2.31) |
| 4 | 1,254 | 50.24 | 20.93 | (7.07, 44.36) | 4.66 | (4.30, 5.05) | 4.53 | (4.18, 4.91) |

**Table S3f: DPT-booster dose, delay if vaccinated after** 24 months **of age**

|  | Total vaccinated | % vaccinated who delay | Median delay from upper limit (wks) | IQR | Crude | 95% CI | Adjusted hazard ratio | 95% CI |
| --- | --- | --- | --- | --- | --- | --- | --- | --- |
| Overall | 9,737 | 11.1 | 16.79 | (6.21, 33.79) |  |  |  |  |
| Year of birth | |  |  |  |  |  |  |  |
| 2005 | 1277 | 17.62 | 26.36 | (10.93, 57.79) | 1 |  | 1 |  |
| 2006 | 2185 | 13.04 | 14.93 | (6.07, 31.64) | 0.85 | (0.78, 0.91) | 0.84 | (0.78, 0.91) |
| 2007 | 2652 | 8.52 | 12.57 | (5.93, 25.07) | 0.73 | (0.68, 0.79) | 0.72 | (0.67, 0.77) |
| 2008 | 1740 | 10.8 | 18.36 | (4.86, 35.71) | 0.71 | (0.66, 0.77) | 0.71 | (0.66, 0.77) |
| 2009 | 1883 | 8.34 | 15.07 | (5.64, 26.93) | 0.64 | (0.59, 0.69) | 0.68 | (0.63, 0.74) |
| Ethnic group | |  |  |  |  |  |  |  |
| Fula | 2,747 | 10.27 | 16.71 | (7.35, 31.64) | 1 |  | 1 |  |
| Mandinka | 2,237 | 10.15 | 17.93 | (5.93, 40.93) | 0.94 | (0.89, 1.00) | 0.99 | (0.93, 1.05) |
| Wollof | 49 | 2.04 | 18.64 | (0.00, 0.00) | 0.74 | (0.55, 0.98) | 0.81 | (0.61, 1.08) |
| Serrehule | 4,551 | 12.35 | 16.00 | (6.07, 33.64) | 1.08 | (1.03, 1.13) | 1.12 | (1.07, 1.18) |
| Other | 129 | 6.2 | 31.79 | (17.36, 36.64) | 0.82 | (0.68, 0.98) | 0.86 | (0.71, 1.03) |
| Gender |  |  |  |  |  |  |  |  |
| Male | 5,044 | 11 | 16.50 | (5.64, 33.07) | 1 |  |  |  |
| Female | 4,693 | 11.21 | 17.43 | (6.93, 34.79) | 1.01 | (0.97, 1.05) |  |  |
| No. previous of delayed vaccinations | | |  |  |  |  |  |  |
| 0 | 6,192 | 6.15 | 13.93 | (5.79, 31.64) | 1 |  | 1 |  |
| 1 | 1,775 | 12.06 | 13.71 | (4.50, 32.93) | 1.52 | (1.43, 1.60) | 1.51 | (1.42, 1.59) |
| 2 | 710 | 15.49 | 18.36 | (6.93, 30.60) | 1.85 | (1.70, 2.01) | 1.85 | (1.70, 2.01) |
| 3 | 499 | 23.25 | 15.79 | (6.57, 31.29) | 2.43 | (2.19, 2.70) | 2.44 | (2.20, 2.71) |
| 4 | 337 | 33.83 | 18.43 | (8.64, 38.93) | 3.12 | (2.73, 3.57) | 3.11 | (2.72, 3.56) |
| 5 | 224 | 65.18 | 24.50 | (12.21, 54.36) | 7.27 | (5.81, 9.09) | 7.10 | (5.66, 8.89) |

**Table S4a: West Kiang, Lower river region: Proportion delayed to vaccination and predictors for delay by vaccine type using the adapted WHO recommended vaccine schedule**

**Table S4a: BCG, delay if vaccinated after 8 weeks of age**

|  | Total vaccinated | % vaccinated who delay | Median delay from upper limit (wks) | IQR | Crude | 95% CI |
| --- | --- | --- | --- | --- | --- | --- |
| Overall | 501 | 2 | 0.78 | (0.29, 2.71) | |  |
| Year of birth | |  |  |  |  |  |
| 2005 | 59 | 1.69 | 0.09 |  | 1 |  |
| 2006 | 93 | 1.08 | 2.14 |  | 0.96 | (0.69, 1.33) |
| 2007 | 103 | 2.91 | 0.14 | (0.14, 2.71) | 1.04 | (0.75, 1.43) |
| 2008 | 97 | 3.09 | 0.71 | (0.57, 6.29) | 1.02 | (0.74, 1.42) |
| 2009 | 98 | 0 |  |  | 0.61 | (0.44, 0.85) |
| 2010 | 51 | 3.92 | 1.57 | (0.29, 2.86) | 0.61 | (0.42, 0.89) |
| Ethnic group | |  |  |  |  |  |
| Fula | 34 | 2.94 | 0.29 |  | 1 |  |
| Mandinka | 451 | 1.77 | 1.50 | (0.43, 2.79) | 0.99 | (0.70, 1.42) |
| Others | 16 | 6.25 | 0.57 |  | 1.08 | (0.59, 1.99) |
| Gender |  |  |  |  |  |  |
| Male | 253 | 1.58 | 0.21 | (0.14, 3.29) | 1 |  |
| Female | 248 | 2.42 | 1.50 | (0.71, 2.71) | 1.02 | (0.85, 1.22) |
|  |  |  |  |  |  |  |

**Table S4b: DPT1, delay if vaccinated after 3 months of age**

|  | Total vaccinated | % vaccinated who delay | Median delay from upper limit (wks) | IQR | Crude | 95% CI |
| --- | --- | --- | --- | --- | --- | --- |
| Overall | 509 | 0.98 | 1.71 | (1.14, 2.00) |  |  |
| Year of birth | |  |  |  |  |  |
| 2005 | 61 | 1.64 | 0.43 | (0.43, 0.43) | 1 |  |
| 2006 | 95 | 0 | 0.00 | (0.00, 0.00) | 0.59 | (0.43, 0.82) |
| 2007 | 106 | 0.94 | 1.71 | (1.71, 1.71) | 0.88 | (0.64, 1.21) |
| 2008 | 98 | 1.02 | 2.14 | (2.14, 2.14) | 0.90 | (0.65, 1.24) |
| 2009 | 99 | 2.02 | 1.57 | (1.14, 2.00) | 1.31 | (0.95, 1.81) |
| 2010 | 50 | 0 | 0.00 | (0.00, 0.00) | 1.11 | (0.76, 1.62) |
| Ethnic group | |  |  |  |  |  |
| Fula | 34 | 2.94 | 1.14 | (1.14, 1.14) | 1 |  |
| Mandinka | 459 | 0.87 | 1.86 | (1.07, 2.07) | 0.78 | (0.55, 1.11) |
| Others | 16 | 0 | 0.00 | (0.00, 0.00) | 0.67 | (0.37, 1.22) |
| Gender |  |  |  |  |  |  |
| Male | 258 | 0.39 | 2.14 | (2.14, 2.14) | 1 |  |
| Female | 251 | 1.59 | 1.43 | (0.79, 1.86) | 1.07 | (0.90, 1.28) |
| BCG vaccination delayed | |  |  |  |  |  |
| 0 | 499 | 0.6 | 1.71 | (1.14, 2.00) | 1 |  |
| 1 | 10 | 20 | 1.29 | (0.43, 2.14) | 3.69 | (1.82, 7.48) |

**Table S4c: DPT2, delay if vaccinated after 5 months of age**

|  | Total vaccinated | % vaccinated who delay | Median delay from upper limit (wks) | IQR | Crude | 95% CI |
| --- | --- | --- | --- | --- | --- | --- |
| Overall | 505 | 0.4 | 14.86 | (0.29, 29.43) |  |  |
| Year of birth | |  |  |  |  |  |
| 2005 | 61 | 0 | 0.00 | (0.00, 0.00) | 1 |  |
| 2006 | 95 | 0 | 0.00 | (0.00, 0.00) | 0.67 | (0.48, 0.92) |
| 2007 | 103 | 0.97 | 0.29 | (0.29, 0.29) | 1.07 | (0.78, 1.47) |
| 2008 | 98 | 0 | 0.00 | (0.00, 0.00) | 1.19 | (0.86, 1.64) |
| 2009 | 98 | 1.02 | 29.43 | (29.43, 29.43) | 1.24 | (0.90, 1.72) |
| 2010 | 50 | 0 | 0.00 | (0.00, 0.00) | 1.11 | (0.76, 1.61) |
| Ethnic group | |  |  |  |  |  |
| Fula | 34 | 2.94 | 0.29 | (0.29, 0.29) | 1 |  |
| Mandinka | 455 | 0.22 | 29.43 | (29.43, 29.43) | 0.77 | (0.54, 1.10) |
| Others | 16 | 0 | 0.00 | (0.00, 0.00) | 0.57 | (0.31, 1.05) |
| Gender |  |  |  |  |  |  |
| Male | 256 | 0.78 | 14.86 | (0.29, 29.43) | 1 |  |
| Female | 249 | 0 | 0.00 | (0.00, 0.00) | 0.98 | (0.82, 1.16) |
| No. previous of delayed vaccinations | | |  |  |  |  |
| 0 | 492 | 0.41 | 14.86 | (0.29, 29.43) | 1 |  |
| 1 | 11 | 0 | 0.00 | (0.00, 0.00) | 2.40 | (1.31, 4.37) |
| 2 | 2 | 0 | 0.00 | (0.00, 0.00) | 5.29 | (1.30, 21.50) |

**Table S4d: DPT3, delay if vaccinated after 7 months of age**

|  | Total vaccinated | % vaccinated who delay | Median delay from upper limit (wks) | IQR | Crude | 95% CI |
| --- | --- | --- | --- | --- | --- | --- |
| Overall | 483 | 5.18 | 2.57 | (1.86, 17.57) |  |  |
| Year of birth | |  |  |  |  |  |
| 2005 | 57 | 3.51 | 2.14 | (1.86, 2.43) | 1 |  |
| 2006 | 94 | 1.06 | 2.57 | (2.57, 2.57) | 0.65 | (0.46, 0.90) |
| 2007 | 99 | 15.15 | 2.14 | (1.14, 10.00) | 1.47 | (1.04, 2.07) |
| 2008 | 95 | 2.11 | 3.14 | (1.86, 4.43) | 1.19 | (0.85, 1.66) |
| 2009 | 91 | 5.49 | 25.71 | (17.57, 50.43) | 1.11 | (0.79, 1.56) |
| 2010 | 47 | 0 | 0.00 | (0.00, 0.00) | 0.89 | (0.60, 1.32) |
| Ethnic group | |  |  |  |  |  |
| Fula | 33 | 3.03 | 2.57 | (2.57, 2.57) | 1 |  |
| Mandinka | 434 | 5.53 | 3.50 | (1.86, 21.64) | 0.85 | (0.59, 1.22) |
| Others | 16 | 0 | 0.00 | (0.00, 0.00) | 0.67 | (0.37, 1.23) |
| Gender |  |  |  |  |  |  |
| Male | 246 | 6.1 | 4.43 | (2.00, 4.43) | 1 |  |
| Female | 237 | 4.22 | 2.29 | (1.14, 9.29) | 1.03 | (0.86, 1.24) |
| No. previous of delayed vaccinations | | |  |  |  |  |
| 0 | 469 | 4.69 | 2.50 | (1.86, 17.57) | 1 |  |
| 1 | 13 | 15.38 | 14.14 | (2.57, 25.71) | 2.35 | (1.29, 4.28) |
| 2 | 1 | 100 | 4.43 | (4.43, 4.43) | 522,000,000 | (287,000,000, 951,000,000) |

**Table S4e: Measles vaccine, delay if vaccinated after 12 months of age**

|  | Total vaccinated | % vaccinated who delay | Median delay from upper limit (wks) | IQR | Crude | 95% CI |
| --- | --- | --- | --- | --- | --- | --- |
| Overall | 452 | 1.33 | 21.43 | (8.36, 46.79) |  |  |
| Year of birth | |  |  |  |  |  |
| 2005 | 56 | 1.79 | 46.79 | (46.79, 46.79) | 1 |  |
| 2006 | 85 | 0 | 0.00 | (0.00, 0.00) | 0.64 | (0.45, 0.90) |
| 2007 | 90 | 0 | 0.00 | (0.00, 0.00) | 1.77 | (1.27, 2.48) |
| 2008 | 91 | 2.2 | 41.07 | (34.50, 47.64) | 2.90 | (2.07, 4.07) |
| 2009 | 88 | 2.27 | 8.36 | (8.36, 8.36) | 2.09 | (1.49, 2.94) |
| 2010 | 42 | 2.38 | 3.21 | (3.21, 3.21) | 1.88 | (1.25, 2.82) |
| Ethnic group | |  |  |  |  |  |
| Fula | 30 | 0 | 0.00 | (0.00, 0.00) | 1 |  |
| Mandinka | 407 | 1.23 | 8.36 | (8.36, 34.50) | 0.94 | (0.65, 1.36) |
| Others | 15 | 6.67 | 47.64 | (47.64, 47.64) | 0.95 | (0.50, 1.79) |
| Gender |  |  |  |  |  |  |
| Male | 235 | 1.7 | 40.64 | (21.43, 47.21) | 1 |  |
| Female | 217 | 0.92 | 5.79 | (3.21, 8.36) | 0.91 | (0.75, 1.09) |
| No. previous of delayed vaccinations | | |  |  |  |  |
| 0 | 419 | 1.43 | 21.43 | (8.36, 46.79) | 1 |  |
| 1 | 29 | 0 | 0.00 | (0.00, 0.00) | 1.36 | (0.93, 1.99) |
| 2 | 3 | 0 | 0.00 | (0.00, 0.00) | 2.72 | (0.87, 8.51) |
| 3 | 1 | 0 | 0.00 | (0.00, 0.00) | 3.61 | (0.51, 25.82) |

**Table S4f: DPT booster dose, delay if vaccinated after 24 months of age**

|  | Total vaccinated | % vaccinated who delay | Median delay from upper limit (wks) | IQR | Crude | 95% CI |
| --- | --- | --- | --- | --- | --- | --- |
| Overall | 0 | 0.61 | 48.29 | (3.64, 92.93) |  |  |
| Year of birth | |  |  |  |  |  |
| 2005 | 50 | 2 | 92.93 | (92.93, 92.93) | 1 |  |
| 2006 | 71 | 0 | 0.00 | (0.00, 0.00) | 1.35 | (0.94, 1.95) |
| 2007 | 73 | 0 | 0.00 | (0.00, 0.00) | 1.63 | (1.13, 2.34) |
| 2008 | 73 | 0 | 0.00 | (0.00, 0.00) | 0.64 | (0.44, 0.92) |
| 2009 | 63 | 1.59 | 3.64 | (3.64, 3.64) | 0.53 | (0.36, 0.77) |
| Ethnic group | |  |  |  |  |  |
| Fula | 21 | 0 | 0.00 | (0.00, 0.00) | 1 |  |
| Mandinka | 297 | 0.67 | 48.29 | (3.64, 92.93) | 1.27 | (0.82, 1.98) |
| Others | 12 | 0 | 0.00 | (0.00, 0.00) | 1.04 | (0.51, 2.12) |
| Gender |  |  |  |  |  |  |
| Male | 172 | 1.16 | 48.29 | (3.64, 92.93) | 1 |  |
| Female | 158 | 0 | 0.00 | (0.00, 0.00) | 0.91 | (0.73, 1.13) |
| No. previous of delayed vaccinations | | |  |  |  |  |
| 0 | 310 | 0.32 | 92.93 | (92.93, 92.93) | 1 |  |
| 1 | 18 | 0 | 0.00 | (0.00, 0.00) | 2.25 | (1.39, 3.63) |
| 2 | 2 | 50 | 3.64 | (3.64, 3.64) | 8.91 | (1.24, 64.03) |

**Table S5a: Western health Region EPI cluster survey: Proportion delayed to vaccination and predictors for delay by vaccine type using the adapted WHO recommended vaccine schedule**

**Table S5a: BCG, delay if vaccinated after 8 weeks of age**

|  | Total vaccinated | % vaccinated who delay | Median delay from upper limit (wks) | IQR | Crude | 95% CI |
| --- | --- | --- | --- | --- | --- | --- |
| Overall | 832 | 6.61 | 4.00 | (1.29, 10.29) |  |  |
| Year of birth | |  |  |  |  |  |
| 2008 | 455 | 8.13 | 5.57 | (1.57, 16.57) | 1 |  |
| 2009 | 377 | 4.77 | 2.14 | (0.86, 7.57) | 0.98 | (0.85, 1.13) |

**Table S5b: DPT1, delay if vaccinated after** 3 months **of age**

|  | Total vaccinated | % vaccinated who delay | Median delay from upper limit (wks) | IQR | Crude | 95% CI |
| --- | --- | --- | --- | --- | --- | --- |
| Overall | 901 | 13.1 | 3.57 | (1.29, 8.71) |  |  |
| Year of birth | |  |  |  |  |  |
| 2008 | 486 | 13.17 | 3.21 | (1.14, 9.93) | 1 |  |
| 2009 | 415 | 13.01 | 3.64 | (2.00, 7.86) | 1.01 | (0.88, 1.16) |
| No. previous of delayed vaccinations | | |  |  |  |  |
| 0 | 837 | 11.59 | 3.29 | (1.14, 7.86) | 1 |  |
| 1 | 54 | 37.04 | 4.86 | (2.07, 12.00) | 2.27 | (1.61, 3.20) |

**Table S5c: DPT2, delay if vaccinated after** 5 months **of age**

|  | Total vaccinated | % vaccinated who delay | Median delay from upper limit (wks) | IQR | Crude | 95% CI |
| --- | --- | --- | --- | --- | --- | --- |
| Overall | 888 | 15.32 | 6.07 | (2.71, 11.93) |  |  |
| Year of birth | |  |  |  |  |  |
| 2008 | 483 | 17.6 | 7.29 | (2.86, 12.71) | 1 |  |
| 2009 | 405 | 12.59 | 5.29 | (2.43, 9.14) | 0.90 | (0.78, 1.04) |
| No. previous of delayed vaccinations | | |  |  |  |  |
| 0 | 742 | 6.6 | 3.86 | (2.43, 9.29) | 1 |  |
| 1 | 127 | 57.48 | 7.29 | (3.00, 12.43) | 5.21 | (3.94, 6.90) |
| 2 | 19 | 73.68 | 7.93 | (2.86, 12.71) | 9.81 | (4.06, 23.68) |

**Table S5d: DPT3, delay if vaccinated after** 7 months **of age**

|  | Total vaccinated | % vaccinated who delay | Median delay from upper limit (wks) | IQR | Crude | 95% CI | Adjusted hazard ratio | 95% CI |
| --- | --- | --- | --- | --- | --- | --- | --- | --- |
| Overall | 840 | 19.29 | 5.93 | (3.14, 15.14) |  |  |  |  |
| Year of birth | |  |  |  |  |  |  |  |
| 2008 | 460 | 23.48 | 6.14 | (3.29, 18.00) | 1 |  | 1 |  |
| 2009 | 380 | 14.21 | 5.86 | (2.71, 10.57) | 0.82 | (0.70, 0.95) | 0.90 | (0.77, 1.04) |
| No. previous of delayed vaccinations | | |  |  |  |  |  |  |
| 0 | 678 | 9.44 | 4.64 | (0.86, 10.93) | 1 |  | 1 |  |
| 1 | 91 | 43.96 | 7.36 | (4.71, 16.86) | 3.33 | (2.50, 4.45) | 3.29 | (2.46, 4.39) |
| 2 | 59 | 83.05 | 8.43 | (4.86, 15.43) | 13.77 | (7.36, 25.78) | 13.73 | (7.33, 25.69) |
| 3 | 12 | 75 | 5.42 | (3.14, 12.00) | 9.67 | (3.11, 30.10) | 9.37 | (3.01, 29.18) |

**Table S5e: Measles vaccine, delay if vaccinated after** 12 months **of age**

|  | Total vaccinated | % vaccinated who delay | Median delay from upper limit (wks) | IQR | Crude | 95% CI |
| --- | --- | --- | --- | --- | --- | --- |
| Overall | 852 | 5.75 | 4.64 | (2.64, 13.50) |  |  |
| Year of birth | |  |  |  |  |  |
| 2008 | 475 | 7.79 | 7.07 | (3.36, 14.21) | 1 |  |
| 2009 | 377 | 3.18 | 3.07 | (2.21, 5.71) | 0.98 | (0.85, 1.12) |
| No. previous of delayed vaccinations | | |  |  |  |  |
| 0 | 616 | 3.25 | 5.57 | (3.57, 14.50) | 1 |  |
| 1 | 117 | 7.69 | 3.21 | (2.64, 7.07) | 1.24 | (1.01, 1.52) |
| 2 | 62 | 16.13 | 6.64 | (1.79, 12.93) | 1.84 | (1.39, 2.45) |
| 3 | 48 | 18.75 | 3.93 | (2.64, 13.50) | 2.05 | (1.48, 2.84) |
| 4 | 9 | 11.11 | 33.07 | (33.07, 33.07) | 1.79 | (0.89, 3.60) |

**Table S6a: Greater Banjul health Clinics: Proportion delayed to vaccination and predictors for delay by vaccine type using the adapted WHO recommended vaccine schedule**

**Table S6a: BCG, delay if vaccinated after 8 weeks of age**

|  | Total vaccinated | % vaccinated who delay | Median delay from upper limit (wks) | IQR | Crude | 95% CI |
| --- | --- | --- | --- | --- | --- | --- |
| Overall | 1,254 | 7.89 | 7.71 | (3.00, 16.00) |  |  |
| Year of birth | |  |  |  |  |  |
| 2007 | 136 | 14.71 | 11.50 | (4.79, 27.93) | 1 |  |
| 2008 | 155 | 12.9 | 8.07 | (3.79, 19.07) | 0.84 | (0.66, 1.08) |
| 2009 | 427 | 4.45 | 5.00 | (1.14, 21.00) | 0.60 | (0.49, 0.74) |
| 2010 | 536 | 7.46 | 7.21 | (2.71, 11.71) | 0.69 | (0.57, 0.85) |
| Ethnic group | |  |  |  |  |  |
| Fula | 225 | 9.78 | 3.71 | (1.29, 10.71) | 1 |  |
| Mandinka | 437 | 8.47 | 8.86 | (3.43, 18.29) | 0.95 | (0.81, 1.13) |
| Wollof | 171 | 5.26 | 6.57 | (4.71, 9.86) | 0.84 | (0.68, 1.03) |
| Jola | 224 | 6.7 | 5.14 | (4.14, 14.14) | 0.82 | (0.68, 1.00) |
| Other | 190 | 8.42 | 11.50 | (4.71, 20.00) | 0.93 | (0.76, 1.13) |
| Gender |  |  |  |  |  |  |
| Male | 644 | 7.45 | 6.50 | (3.07, 13.50) | 1 |  |
| Female | 608 | 8.39 | 8.00 | (3.00, 18.29) | 1.04 | (0.92, 1.17) |

**Table S6b: DPT1, delay if vaccinated after** 3 months **of age**

|  | Total vaccinated | % vaccinated who delay | Median delay from upper limit (wks) | IQR | Crude | 95% CI |
| --- | --- | --- | --- | --- | --- | --- |
| Overall | 1,400 | 7.93 | 4.14 | (1.71, 10.00) |  |  |
| Year of birth | |  |  |  |  |  |
| 2007 | 153 | 8.5 | 7.00 | (3.29, 36.43) | 1 |  |
| 2008 | 172 | 9.88 | 4.43 | (4.00, 12.57) | 0.91 | (0.72, 1.15) |
| 2009 | 470 | 8.3 | 3.43 | (0.86, 6.14) | 0.92 | (0.76, 1.11) |
| 2010 | 605 | 6.94 | 3.93 | (1.71, 9.29) | 0.85 | (0.71, 1.03) |
| Ethnic group | |  |  |  |  |  |
| Fula | 246 | 7.32 | 5.29 | (2.29, 10.29) | 1 |  |
| Mandinka | 493 | 8.11 | 4.00 | (2.36, 9.29) | 1.02 | (0.87, 1.20) |
| Wollof | 195 | 7.18 | 3.93 | (0.86, 7.00) | 0.94 | (0.78, 1.15) |
| Jola | 243 | 8.23 | 4.29 | (1.43, 11.43) | 1.07 | (0.89, 1.29) |
| Other | 214 | 8.88 | 3.86 | (1.43, 10.00) | 1.05 | (0.86, 1.27) |
| Gender |  |  |  |  |  |  |
| Male | 727 | 8.94 | 4.14 | (1.57, 9.29) | 1 |  |
| Female | 671 | 6.86 | 4.29 | (2.29, 10.00) | 1.02 | (0.92, 1.14) |
| Delayed BCG vaccination | |  |  |  |  |  |
| No | 1,285 | 4.82 | 3.43 | (0.86, 6.86) | 1 |  |
| Yes | 98 | 50 | 7.00 | (3.29, 12.71) | 4.31 | (3.23, 5.75) |

**Table S6c: DPT2, delay if vaccinated after** 5 months **of age**

|  | Total vaccinated | % vaccinated who delay | Median delay from upper limit (wks) | IQR | Crude | 95% CI |
| --- | --- | --- | --- | --- | --- | --- |
| Overall | 1,400 | 11.86 | 4.86 | (1.43, 9.00) |  |  |
| Year of birth | |  |  |  |  |  |
| 2007 | 153 | 14.38 | 5.64 | (2.14, 11.00) | 1 |  |
| 2008 | 172 | 13.95 | 8.71 | (4.86, 15.86) | 0.93 | (0.74, 1.18) |
| 2009 | 470 | 12.55 | 3.42 | (1.29, 8.57) | 0.87 | (0.72, 1.06) |
| 2010 | 605 | 10.08 | 4.57 | (1.14, 8.00) | 0.83 | (0.68, 1.00) |
| Ethnic group | |  |  |  |  |  |
| Fula | 246 | 11.79 | 5.00 | (1.29, 7.71) | 1 |  |
| Mandinka | 493 | 11.36 | 4.93 | (1.43, 8.57) | 0.97 | (0.82, 1.14) |
| Wollof | 195 | 12.82 | 3.86 | (2.00, 8.00) | 0.99 | (0.81, 1.21) |
| Jola | 243 | 14.4 | 5.57 | (0.57, 16.43) | 1.07 | (0.88, 1.29) |
| Other | 214 | 9.81 | 5.86 | (2.71, 11.29) | 0.97 | (0.80, 1.18) |
| Gender |  |  |  |  |  |  |
| Male | 727 | 13.2 | 4.29 | (1.43, 8.43) | 1 |  |
| Female | 671 | 10.28 | 6.43 | (2.00, 12.14) | 0.96 | (0.86, 1.08) |
| No. previous of delayed vaccinations | | |  |  |  |  |
| 0 | 1,240 | 6.53 | 2.57 | (0.86, 6.86) | 1 |  |
| 1 | 111 | 44.14 | 4.57 | (2.29, 11.43) | 3.64 | (2.81, 4.71) |
| 2 | 49 | 73.47 | 8.57 | (6.43, 16.86) | 9.85 | (5.69, 17.04) |

**Table S6d: DPT3, delay if vaccinated after** 7 months **of age**

|  | Total vaccinated | % vaccinated who delay | Median delay from upper limit (wks) | IQR | Crude | 95% CI |
| --- | --- | --- | --- | --- | --- | --- |
| Overall | 1,389 | 15.55 | 6.29 | (2.86, 12.26) |  |  |
| Year of birth | |  |  |  |  |  |
| 2007 | 152 | 16.45 | 6.57 | (3.00, 16.57) | 1 |  |
| 2008 | 171 | 18.13 | 9.57 | (2.57, 22.29) | 1.11 | (0.87, 1.41) |
| 2009 | 465 | 16.34 | 8.00 | (3.57, 12.93) | 0.96 | (0.78, 1.17) |
| 2010 | 601 | 13.98 | 4.50 | (2.79, 7.79) | 0.95 | (0.78, 1.15) |
| Ethnic group | |  |  |  |  |  |
| Fula | 244 | 14.75 | 6.36 | (3.57, 12.14) | 1 |  |
| Mandinka | 487 | 14.37 | 5.79 | (3.00, 10.43) | 0.93 | (0.79, 1.10) |
| Wollof | 195 | 14.87 | 6.29 | (3.86, 12.14) | 0.96 | (0.79, 1.18) |
| Jola | 243 | 18.11 | 8.36 | (2.71, 20.71) | 1.01 | (0.83, 1.23) |
| Other | 211 | 17.06 | 5.86 | (2.29, 11.79) | 1.01 | (0.82, 1.23) |
| Gender |  |  |  |  |  |  |
| Male | 720 | 17.08 | 6.29 | (2.86, 12.00) | 1 |  |
| Female | 667 | 13.79 | 6.21 | (2.93, 14.07) | 0.93 | (0.83, 1.04) |
| No. previous of delayed vaccinations | | |  |  |  |  |
| 0 | 1,156 | 6.92 | 3.93 | (2.00, 7.64) | 1 |  |
| 1 | 138 | 46.38 | 6.29 | (3.36, 12.00) | 3.84 | (3.03, 4.87) |
| 2 | 60 | 66.67 | 9.29 | (4.64, 20.50) | 7.45 | (4.78, 11.62) |
| 3 | 35 | 91.43 | 10.21 | (4.71, 21.86) | 31.84 | (10.24, 98.99) |

**Table S6e: Measles vaccine, delay if vaccinated after** 12 months **of age**

|  | Total vaccinated | % vaccinated who delay | Median delay from upper limit (wks) | IQR | Crude | 95% CI | Adjusted hazard ratio | 95% CI |
| --- | --- | --- | --- | --- | --- | --- | --- | --- |
| Overall | 1,345 | 7.06 | 8.79 | (4.21, 25.79) |  |  |  |  |
| Year of birth | |  |  |  |  |  |  |  |
| 2007 | 150 | 14 | 9.50 | (3.36, 35.50) | 1 |  | 1 |  |
| 2008 | 167 | 10.78 | 10.21 | (4.21, 23.21) | 0.93 | (0.73, 1.18) | 0.87 | (0.69, 1.11) |
| 2009 | 469 | 8.53 | 12.21 | (4.71, 29.00) | 0.82 | (0.67, 0.99) | 0.82 | (0.67, 1.00) |
| 2010 | 559 | 2.86 | 4.36 | (2.79, 8.21) | 0.63 | (0.52, 0.76) | 0.65 | (0.53, 0.79) |
| Ethnic group | |  |  |  |  |  |  |  |
| Fula | 232 | 8.19 | 8.50 | (4.21, 29.07) | 1 |  |  |  |
| Mandinka | 477 | 5.87 | 12.14 | (5.29, 34.14) | 1.03 | (0.87, 1.21) |  |  |
| Wollof | 185 | 5.95 | 5.93 | (1.36, 13.50) | 1.00 | (0.82, 1.22) |  |  |
| Jola | 237 | 8.86 | 11.79 | (2.93, 24.21) | 1.15 | (0.95, 1.39) |  |  |
| Other | 207 | 7.25 | 5.93 | (3.79, 15.64) | 1.00 | (0.83, 1.22) |  |  |
| Gender |  |  |  |  |  |  |  |  |
| Male | 699 | 8.44 | 8.36 | (4.07, 25.79) | 1 |  |  |  |
| Female | 645 | 5.58 | 10.79 | (4.21, 26.36) | 0.93 | (0.83, 1.04) |  |  |
| No. previous of delayed vaccinations | | |  |  |  |  |  |  |
| 0 | 1,044 | 4.12 | 6.50 | (3.36, 29.50) | 1 |  | 1 |  |
| 1 | 153 | 9.8 | 9.50 | (2.93, 22.93) | 1.58 | (1.32, 1.89) | 1.58 | (1.32, 1.89) |
| 2 | 77 | 14.29 | 8.79 | (2.64, 19.64) | 1.78 | (1.39, 2.29) | 1.72 | (1.34, 2.21) |
| 3 | 42 | 28.57 | 11.29 | (8.36, 27.43) | 2.80 | (1.95, 4.03) | 2.70 | (1.88, 3.90) |
| 4 | 29 | 48.28 | 7.36 | (4.50, 25.79) | 4.44 | (2.66, 7.41) | 4.25 | (2.54, 7.09) |
